# Hemagglutinin Nanoparticulate Vaccine with Controlled Photochemical Immunomodulation for Pathogenic Influenza‐Specific Immunity

**DOI:** 10.1002/advs.202100118

**Published:** 2021-10-24

**Authors:** Hayoon Jeong, Chung‐Sung Lee, Jangsu Lee, Jonghwan Lee, Hee Sook Hwang, Min Lee, Kun Na

**Affiliations:** ^1^ Department of Biomedical‐Chemical Engineering The Catholic University of Korea Bucheon‐si Gyeonggi‐do 14662 Republic of Korea; ^2^ Department of Biotechnology The Catholic University of Korea Bucheon‐si Gyeonggi‐do 14662 Republic of Korea; ^3^ Division of Advanced Prosthodontics University of California Los Angeles Los Angeles CA 90095 USA; ^4^ Department of Pharmaceutical Engineering and Biotechnology Sun Moon University Asan‐si Chungcheongnam‐do 31460 Republic of Korea; ^5^ Department of Pharmaceutical Engineering Dankook University Cheonan‐si Chungcheongnam‐do 31116 Republic of Korea; ^6^ Department of Bioengineering University of California Los Angeles Los Angeles CA 90095 USA

**Keywords:** influenza virus, nanovaccine, nasal antigen delivery, nasal vaccine, photochemical immunomodulation

## Abstract

Recently, viral infectious diseases, including COVID‐19 and Influenza, are the subjects of major concerns worldwide. One strategy for addressing these concerns focuses on nasal vaccines, which have great potential for achieving successful immunization via safe, easy, and affordable approaches. However, conventional nasal vaccines have major limitations resulting from fast removal when pass through nasal mucosa and mucociliary clearance hindering their effectiveness. Herein a nanoparticulate vaccine (NanoVac) exhibiting photochemical immunomodulation and constituting a new self‐assembled immunization system of a photoactivatable polymeric adjuvant with influenza virus hemagglutinin for efficient nasal delivery and antigen‐specific immunity against pathogenic influenza viruses is described. NanoVac increases the residence period of antigens and further enhances by spatiotemporal photochemical modulation in the nasal cavity. As a consequence, photochemical immunomodulation of NanoVacs successfully induces humoral and cellular immune responses followed by stimulation of mature dendritic cells, plasma cells, memory B cells, and CD4^+^ and CD8^+^ T cells, resulting in secretion of antigen‐specific immunoglobulins, cytokines, and CD8^+^ T cells. Notably, challenge with influenza virus after nasal immunization with NanoVacs demonstrates robust prevention of viral infection. Thus, this newly designed vaccine system can serve as a promising strategy for developing vaccines that are active against current hazardous pathogen outbreaks and pandemics.

## Graphical abstract

A hemagglutinin nanoparticulate vaccine (NanoVac) exhibiting photochemical immunomodulation as a new immunization system is developed for efficient antigen‐specific immunity against pathogenic influenza viruses. The NanoVac increases the residence period of antigens by photochemical modulation effected with spatiotemporal light exposure. The NanoVac with controlled photochemical immunomodulation successfully induces humoral and cellular immune responses, resulting in robust prevention of viral infection.

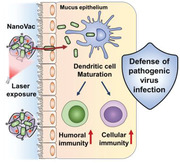

## 1 Introduction

Infectious diseases, including coronavirus disease (COVID‐19) and Influenza, are major causes for concern worldwide, and the emergence of novel viral strains, including influenza virus and coronavirus, has resulted in epidemics or pandemics. Several viral infectious diseases are not subject to immediate and effective prophylactic vaccination. There is, therefore, an urgent need for the development of a quick, safe, and efficient vaccine strategy for preventing and surmounting viral infections.

Since the respiratory system and particularly the nasal passages are the primary site infected by most pathogens, nasal vaccines have attracted considerable interest in the treatment of infectious diseases.^[^ ^1^, ^2^ ^]^ Mucosal immunization has strong potential for inducing concurrent cellular and humoral immune responses via stimulation of nasal‐associated lymphoid tissues.^[^ ^3^, ^4^, ^5^, ^6^, ^7^, ^8^ ^]^ In comparison with parenteral administration of systemic vaccines, nasal vaccines are much simpler to manufacture and it is easier to control dosage and administration.^[^ ^2^, ^4^, ^6^, ^9^ ^]^ In addition, nasal administration is noninvasive, convenient, poses little risk from blood‐borne infections, and exhibits increased patient compliance because there is no need for the use of needles.^[^ ^10^, ^11^, ^12^, ^13^, ^14^ ^]^ However, conventional nasal vaccines are hindered by highly viscoelastic and sticky mucus layers, which act as barriers to the delivery of antigens exhibiting a low affinity for mucosal surfaces, fast clearance, and a significant decrease in the passage of the high‐molecular‐weight antigens (greater than 1000 Da).^[^ ^4^, ^15^, ^16^, ^17^ ^]^ The major challenge for the success of mucosal vaccines results from nasal delivery to target antigen‐presenting cells or tissues, which is required for efficient induction of immune responses.

Particulate antigen delivery systems have received increasing interest because of their effectiveness for the protection of antigens against proteolytic degradation in the nasal mucosa.^[^ ^1^, ^18^, ^19^, ^20^, ^21^ ^]^ Subcomponent‐based mucosal vaccines with nonviral nanodelivery systems, consisting of mucoadhesive or cationic polymers, cationic peptides, dendrimers, and lipid compounds, have been developed to minimize mucociliary clearance, promote penetration efficiency, and interact with immune cells, thereby triggering efficient antigen‐specific immunity.^[^ ^21^, ^22^, ^23^, ^24^, ^25^, ^26^, ^27^ ^]^ However, their poor immunostimulatory properties lead to vaccination efficacies lower than those seen with viral vaccines.^[^ ^1^, ^4^, ^15^, ^21^, ^27^ ^]^ Therefore, there is a need to enhance the immunogenicity of antigens, and, as a consequence, vaccination efficacy.^[^ ^28^, ^29^, ^30^ ^]^ Adjuvants are synthetic and natural compounds with different origins and endogenous substances that are identified as immunomodulators.^[^ ^31^, ^32^, ^33^ ^]^ The uses of adjuvants in vaccine formulations involve designs with selective induction or alteration of the immune responses and controlled release of antigens.^[^ ^1^, ^21^, ^31^, ^34^ ^]^

**Figure 1:**
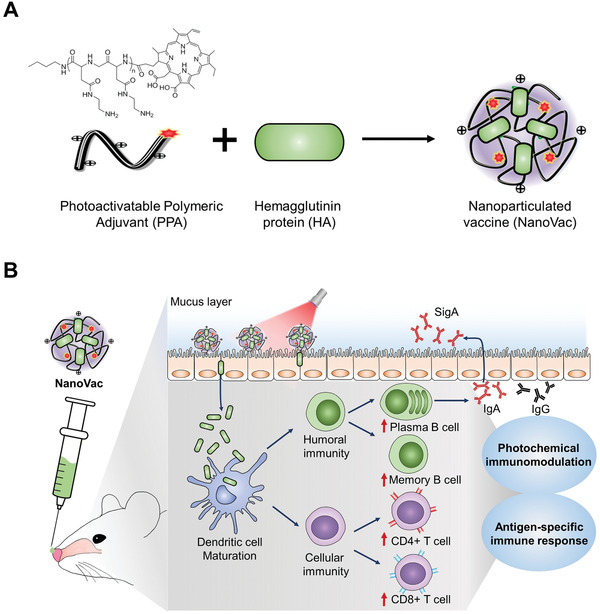
Formulation and immunomodulation process of NanoVac. A) Nanoparticulate vaccine (NanoVac) was composed of photoactivatable polymeric adjuvant (PPA) and influenza hemagglutinin protein (HA) as an antigen. B) Schematic illustration of photochemical immunomodulation according to intranasally administration of NanoVac and laser irradiation. Vaccine delivery via nanoformulation induces antigens to increase the residence time in nasal cavity. Photochemical immunomodulation of NanoVac was induced dendritic cell maturation and humoral immune response effectively and further cellular immunity. In consequence of photoactivatable adjuvancity effect, antibodies were well produced such as IgG and IgA. (HA, hemagglutinin; IgG, immunoglobulin G; IgA, immunoglobulin A; SigA, secretory IgA).

We present herein a nanoparticulate vaccine (NanoVac) with photochemical immunomodulation exhibiting efficient nasal antigen delivery and antigen‐specific immunity to pathogenic viruses (**Figure** **1**). The NanoVac was readily prepared via electrostatic self‐assembly of photoactivatable polymeric adjuvant (PPA) and high molecular weight antigens such as ovalbumin (OVA) or hemagglutinin (HA) as model antigens. For the PPA, poly[(2‐aminoethyl)aspartamide]‐chlorin e6 was synthesized using poly(ʟ‐aspartic acid) and photosensitizer chlorin e6 (Ce6). The partial amination of the carboxyl groups of aspartic acid chains can increase mucoadhesive properties and residence times, which leads to enhanced antigen delivery through interactions with the negatively charged epithelial cell surface.^[^ ^35^, ^36^, ^37^, ^38^, ^39^ ^]^ The Ce6 photosensitizing molecule as a photochemical immunomodulator was used to generate reactive oxygen species (ROS) for enhancing mucus barrier penetration and stimulating immune responses.^[^ ^40^, ^41^ ^]^ The photochemical activity of this and related photosensitizers has been shown to improve tissue penetration efficiency and increase photochemical internalization with cellular and tissue barriers.^[^ ^42^, ^43^ ^]^ In addition, photochemical adjuvanticity has also been demonstrated to induce immune responses by stimulating the expression of transcription factors that control both immunoregulatory proteins and inflammatory cytokines.^[^ ^44^, ^45^, ^46^, ^47^, ^48^ ^]^ In the present study, NanoVac integrated with the photochemical immunomodulation showed a higher induction of humoral and cellular immunity and provides new insights into the pathogenesis of the infection and improvements in treatment options.

## 2 Results and Discussion

### 2.1 Formulation of NanoVac

**Figure 2:**
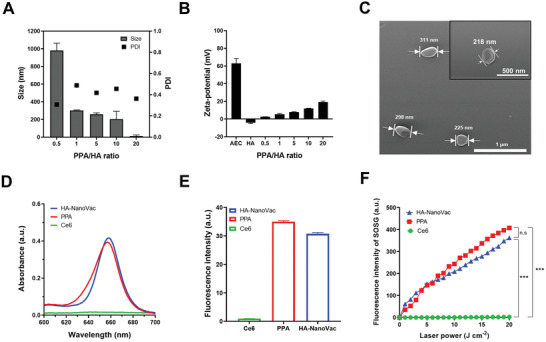
Characterization of HA‐NanoVac. A) Particle size and PDI value of NanoVac on difference of PPA/HA weight ratio. B) Zeta‐potential of HA‐NanoVac. C) Field emission‐scanning electron microscope (FE‐SEM) image of HA‐NanoVac at a PPA/HA weight ratio of 5. D) The UV–vis spectra of Ce6, PPA, and HA‐NanoVac in D.I. water. E) Fluorescence intensity of Ce6, PPA, and HA‐NanoVac at 663 nm. F) Fluorescence intensity of singlet oxygen sensor green (SOSG) with Ce6, PPA, and HA‐NanoVac were measured at 534 nm (excitation 494 nm, emission 534 nm). Laser irradiation at 671 nm (*** *P* < 0.001, ns, not significant, one‐way ANOVA, and Tukey multiple comparison test).

PPA was synthesized via a ring‐opening polymerization and carbodiimide reaction using poly(ʟ‐aspartic acid), which is known as a highly anionic and biocompatible polymer, as the backbone polymer (Figure S1, Supporting Information).^[^ ^44^, ^49^, ^50^, ^51^ ^]^ The PPA was synthesized via an aminolysis reaction to make a polymer bearing positive charges. The chemical structures of poly(*β*‐benzyl‐ʟ‐aspartate) (PBLA), poly(*β*‐benzyl‐ʟ‐aspartate)‐chlorin e6 (PBLA‐Ce6), and PPA were confirmed using ^1^H‐NMR (Figures S2A, S3, and S4, Supporting Information). PBLA was further analyzed by gel permeation chromatography (GPC) to determine the weight‐average molecular weight (Mw: 12 480), the number‐average molecular weight (Mn: 7900), and the polydispersity index (PDI: 1.58) (Figure S2B, Supporting Information). NanoVacs were formulated by mixing PPA with influenza hemagglutinin (HA) protein antigen. A complex structure was readily achieved from ionic and hydrophobic interactions between cationic PPA and anionic HA in the physiological pH range. During the mixing process, different PPA/HA weight ratios were used to identify the complex exhibiting optimal colloidal and structural stability, as determined with the use of dynamic light scattering (DLS). NanoVacs exhibited an average size of ≈240 nm and PDI values ranging between 0.2 and 0.3 (**Figure** **2A**). As PPA ratio of the NanoVac increased, the zeta‐potential was also increased (Figure 2B). We chose HA‐NanoVac with a PPA/HA weight ratio of 5 for further study. Field emission‐scanning electron microscopy (FE‐SEM) imaging showed that HA‐NanoVacs were 200–300 nm in size, with a round shape and a smooth surface morphology (Figure 2C).

To evaluate the photoactivity of NanoVacs, the UV–vis spectra and fluorescence intensities of Ce6, PPA, and HA‐NanoVacs were compared. The UV–vis spectra of Ce6 and PPA were identical (Figure S5, Supporting Information). The Ce6 content of the PPA conjugates was determined from the absorption at 663 nm. The UV–vis spectrum of HA‐NanoVac is nearly identical to that of PPA in deionized (D.I.) water (Figure 2D), and fluorescence intensities at 663 nm were also quite similar (Figure 2E). Additionally, we performed a singlet oxygen generation test using singlet oxygen sensor green (SOSG). There were similar levels of singlet oxygen produced by PPA and HA‐NanoVac (Figure 2F). Free Ce6 exhibited reduced photoactivity due to an aqueous quenching effect resulting from poor solubility and consequent aggregation occurring via hydrophobic interactions.^[^ ^52^ ^]^ The optical properties of the NanoVacs were analogous to those of PPA, including the UV–vis spectra, fluorescence intensities, and levels of singlet oxygen generation. Thus, the optical properties were retained upon formulation of NanoVac with HA.

Additionally, photo‐mediated activation of PPA was confirmed with the use of a photomultiplier (PMT) detector with three bandpass filters, using a µJ pulse of 671 nm irradiation and the subsequent detection of the luminescence from singlet oxygen (SO). With 5 µs pulse duration, PPA generated luminescence signals for a period of ≈25 µs (Figure S6, Supporting Information).

Furthermore, it was confirmed that the preparation of NanoVac was readily performed using other proteins, such as ovalbumin (OVA). OVA has been used as an antigenic model protein in various studies. The PI (isoelectric point) value for OVA is 4.7 and it bears a negative charge in the physiological pH range.^[^ ^53^, ^54^ ^]^ OVA‐NanoVac was formulated using the procedure described above. OVA‐NanoVac exhibited an average size of ≈240 nm and had a zeta‐potential of ≈70 mV and PDI values between 0.2 and 0.3. FE‐SEM studies on OVA‐NanoVacs confirmed that they had a similar size and a round shape (Figure S7A–C, Supporting Information). Singlet oxygen generation by OVA‐NanoVac was confirmed using a PMT detector with three bandpass filters. With a 5 µs pulse duration, OVA‐NanoVac successfully generated signals over a period of 25 µs (Figure S7D, Supporting Information). All of these results indicated tendencies similar to those of HA‐NanoVac.

Next, we confirmed a structural and antigenic integrities of HA protein in NanoVac formulations using circular dichroism (CD) spectroscopy (Figure S8, Supporting Information), sodium dodecyl sulfate‐polyacrylamide gel electrophoresis (SDS‐PAGE), and western blot (Figure S9, Supporting Information). CD analysis suggested that there was no significant difference between HA and HA‐NanoVac spectrum even when it is formulated with PPA and laser irradiation. As shown in gel electrophoresis, band thicknesses of HA proteins for NanoVac formulations have no significant difference in the presence or absence of laser irradiation at 1 J cm^−2^ (Figure S9A,B, Supporting Information). Additionally, similar results were shown in the western blot performed with anti‐HA specific antibodies (Figure S9C,D, Supporting Information). Thus, these results supported that the antigenic HA in the NanoVac system can safely formulate and deliver, even under photochemical stimulation, maintaining their structural stabilities and antigenic integrities.

### 2.2 Increased Antigen Residence Time of Vaccine Formulation in the Nasal Cavity

**Figure 3:**
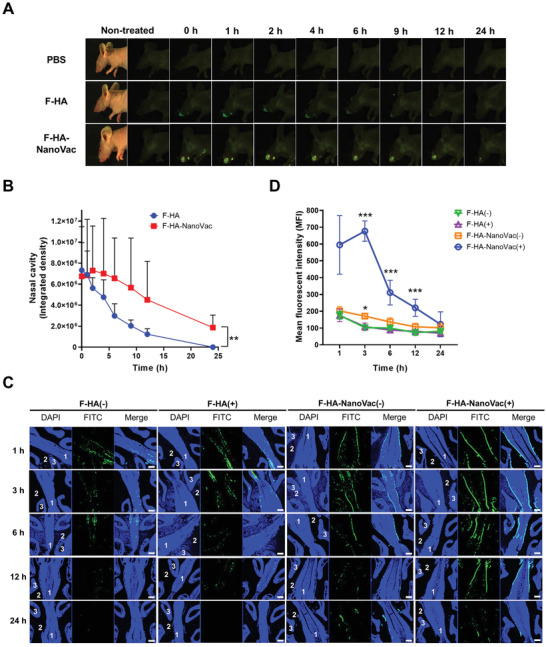
Particulate nanovaccine delivery formulation increased residence time in nasal cavities. A) Fluorescence images by time after administered intranasally of PBS, FITC labeled HA (F‐HA), and F‐HA‐NanoVac (FOBI, Neo Science, Korea). Fluorescence images of mice were captured up to 24 h after administration. B) Fluorescence intensity in nasal area until 24 h after inoculation were compared for each mouse group (BALB/c nude, male, 6 weeks, *n* = 3) (** *P* < 0.01, Student's *t*‐test comparison). C) HA protein residence time in the nasal cavity was determined after intranasal administration with F‐HA and F‐HA‐NanoVac that endured presence (+) or absence (−) of laser irradiation. Fluorescence images of the nasal cavity of mice were observed by confocal laser scanning microscopy. Histological indication of mouse nasal cavity with white numbers (1–3). 1, nasal septum; 2, nasal meatus; 3, nasal turbinate. Scale bar: 200 µm. D) Mean fluorescence intensity in nasal septum of (C) (two‐way ANOVA and Tukey multiple comparison test, * *P* < 0.05, and *** *P* < 0.001).

It is necessary to increase the residence time of antigens in intranasal vaccination because of the nasal mucociliary clearance system.^[^ ^55^, ^56^ ^]^ HA protein was labeled with fluorescein isothiocyanate (FITC) to verify whether the residence time in nasal cavity was increased by the NanoVac formulation. First, the FITC‐labeled HA (F‐HA) and F‐HA‐NanoVac were administered to mice intranasally (BALB/c nude, male, 6 weeks, *n* = 3), and then fluorescence images were captured at various times using a fluorescence‐labeled organism bioimaging instrument (FOBI, Neo Science, Korea) (**Figure** **3A**). It was shown that the intensity of green fluorescence at the nose region was increased in the group to which NanoVac was administrated. Comparisons run for 24 h after administration showed that the fluorescence seen with the F‐HA‐NanoVac treated mice exhibited greater intensity and lasted longer than did the fluorescence seen with F‐HA administered mice. The fluorescence intensities of F‐HA and F‐HA‐NanoVac gradually decreased over time (Figure 3B). Comparing the fluorescence intensities after 12 h shows that the intensity of F‐HA‐NanoVac emission was 3.7‐fold higher than that of free F‐HA. When free F‐HA was delivered, the fluorescence had completely disappeared 24 h after administration. However, the fluorescence in the NanoVac group was continuously detected for up to 24 h after administration. Thus, it was confirmed that the NanoVac formulations with antigenic proteins, including HA and OVA, exhibited prolonged residence times in the nasal cavity.

To observe the intranasal distribution of antigenic protein and formulated NanoVac, sectionalized nasal tissues were analyzed using confocal microscopy (Figure 3C; Figure S11A, Supporting Information). After intranasal administration of F‐HA and F‐HA‐NanoVac to mice, nasal tissue was dissected at various time points. When comparing F‐HA and F‐HA‐NanoVacs in the absence of laser irradiation, the fluorescence intensity resulting from F‐HA‐NanoVac in the nasal cavity was greater than that of the free F‐HA group. Additionally, the highest fluorescence intensity was detected in F‐HA‐NanoVac administered and laser‐irradiated mice. Fluorescence intensity was not detected past 12 h for nasal cavities treated with F‐HA, whereas fluorescence intensity was observed for 24 h after in NanoVac administered and laser‐irradiated mice (Figure 3D). This tendency is similar to that showed when using a nanoparticulate formulation with OVA protein (OVA‐NanoVac). When the FITC‐labeled OVA (F‐OVA)‐NanoVac formulation was delivered, fluorescence intensity in nasal tissues was increased and the fluorescence signal was maintained for a longer time, relative to those showed after administration of free F‐OVA (Figure S11B, Supporting Information). After administration and laser irradiation of the nasal region, administration of F‐HA‐NanoVac in nasal tissue was showed higher fluorescence intensity in the nasal septum than was shown with F‐HA; this was due to the photoactivity of the PPA in the NanoVac formulation. When F‐HA‐NanoVac was administered intranasally to mice and laser irradiation, ROS can be generated locally by the photoactivity of photosensitizer, Ce6, in the PPA. As previously reported, ^[^ ^43^, ^57^, ^58^ ^]^ photochemical reaction by the ROS generated from the photosensitizer can increase the tissue penetration of nanoparticles. Based on this mechanism, we expected that the photoactivity of NanoVac system can enhance the penetration of nasal mucus layer, which is an outermost layer of the nasal tissue. Thus, nanoparticulate formulations of antigenic proteins with PPA enhanced the residence times in the nasal cavity. Notably, laser irradiation after NanoVac administration allows to increase the amount of antigen in the nasal cavity as a result of the photoactivity of NanoVac.

### 2.3 Photochemical Induction of Immune Response with Intranasal Administration of NanoVac

**Figure 4:**
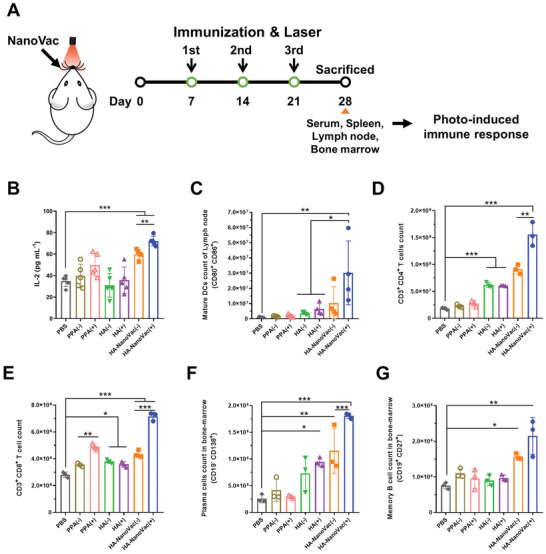
Photochemical immune induction of administration of NanoVac into nasal cavities. A) Schematic illustration of immunization and sample collection strategies. B) Confirmation of IL‐2 cytokine levels in serum collected by submandibular after immunization via ELISA kit (BALB/c, male, 6 weeks, *n* = 5). C–G) FACS analysis of cellular immunity and humoral immunity in immunization and laser irradiated mice. (C) Dendritic cell maturation according to immunization and laser irradiation (CD11c^+^ and CD80^+^/CD86^+^ cells in lymph node, BALB/c, male, 6 weeks, *n* = 4). (D) CD4^+^ helper T cells and (E) CD8^+^ cytotoxic T cells were isolated from spleen of immunized mice (CD3^+^/CD4^+^ T cells and CD3^+^/CD8^+^ T cells in spleen, BALB/c, male, 6 weeks, *n* = 3). (F) Plasma cells and (G) memory B cells were detected in bone marrow of immunized mice (CD19^−^/CD138^+^ plasma cells and CD19^+^/CD27^+^ memory B cells in bone marrow of mouse femur, BALB/c, male, 6 weeks, *n* = 3). Statistical significance was determined using two‐way ANOVA and Dunnett's multiple comparison tests by GraphPad Prism. Error bars represent the means ± SD (* *P* < 0.05, ** *P* < 0.01, and *** *P* < 0.001).

To identify the induction of photochemical immune responses, PPA, HA protein, and HA‐NanoVac were delivered intranasally for immunization in the presence or absence of laser irradiation (**Figure** **4A**). After three times of immunization were completed, the concentration of interleukin‐2 (IL‐2), CC motif chemokine ligand 3 (CCL3) and CCL4 cytokines in the serum of the mice were measured by enzyme‐linked immunosorbent assay (ELISA) analysis (Figure 4B; Figure S12, Supporting Information). IL‐2 is a Th1 type cytokine and is well known to induce a proinflammatory immune response.^[^ ^59^, ^60^, ^61^ ^]^ CCL3 and CCL4 cytokines were induced CD8^+^ T cells immunity that effectively could defense against influenza virus infections. After immunization, the IL‐2 concentration of HA‐NanoVac group in serum was ≈1.8‐fold higher than was that shown with delivery of the free HA group. When immunization was performed using HA‐NanoVac and then laser irradiation, the IL‐2 cytokine level was 2.2‐fold higher than that shown with the free HA group. This level was ≈1.2‐fold higher than that of the NanoVac without laser irradiation. Additionally, the CCL3 and CCL4 cytokine levels of immunized mice using HA‐NanoVac and laser irradiation were increased 5% and 20% than that shown with the free HA group (Figure S12, Supporting Information). The NanoVac system effectively induced an immune response and photochemical immunomodulation via laser irradiation showed an enhanced level of immune response.

In order to increase the effectiveness of the vaccine, it is important to induce the maturation of dendritic cells (DCs) following antigen delivery.^[^ ^62^, ^63^, ^64^ ^]^ To confirm the immune inducing effect of NanoVac and laser irradiation, the developed HA‐NanoVac was treated to DC cells, which are one of the representative antigen presenting cells (Figure S13, Supporting Information). DC cells were isolated from mouse bone marrow and then cultured to obtain immature DCs. Theses immature bone marrow derived DCs (BMDCs) were treated with HA‐NanoVac in the presence or absence of laser irradiation. The number of mature DCs was detected by flow cytometry analysis using CD11c, CD80, and CD86 markers (Table S2, Supporting Information). CD80 and CD86 molecules in DCs serve to elevate immunomodulation of Th1 cells (helper T cells, CD4^+^ T cells) response.^[^ ^65^, ^66^, ^67^, ^68^ ^]^ When antigen protein (HA) was exposed to immature DCs using the NanoVac system, the number of mature DCs were increased to 16% compared to free HA‐treated group. In HA‐NanoVac treated and laser irradiations, the number of mature DCs increased about 43% compared to the free HA‐treated DCs. Notably, the PPA‐treated DCs were not significantly different from the phosphate buffered saline (PBS)‐treated DCs, but when irradiated with a laser after PPA treatment, the number of mature DCs was increased about 60% compared to the PBS‐treated DCs. With the laser irradiation after HA‐NanoVac treatment, the number of mature DCs was increased 87% compared to PBS‐treated group. Therefore, the delivery of the nanoparticulated antigen via NanoVac increased the number of mature DCs in the in vitro condition. Particularly, the number of mature DCs in the presence of laser increased drastically due to the photochemical effect of the PPA of NanoVac. The lymph nodes of immunized mice were dissected and homogenized to detect the level of maturation of DC cells (Figure 4C; Figure S14, Supporting Information). The BMDCs were isolated to confirm the maturation that immunized mice via NanoVac formulation and laser irradiation (Figure S15, Supporting Information). Immunization with NanoVac formulations increased the number of mature DCs approximately twofold, relative to delivery of antigen protein alone. In mice immunized with NanoVac, the use of laser irradiation resulted in more mature DCs than was shown in the absence of laser irradiation. When immunized with NanoVac and irradiated, levels of mature DCs increased to a level approximately ninefold greater than that shown with only HA protein delivered, and threefold greater than that shown without laser irradiation of mice immunized with NanoVac. The use of NanoVac as a vaccine delivery system increases DC maturation and causes effective induction of immune response.

The activity of helper T cells is essential for B cell upregulation in the immune response against antigens.^[^ ^69^, ^70^ ^]^ The number of helper T cells (CD4^+^ T cells) was confirmed by flow cytometry analysis using CD3 and CD4 markers in the spleens of immunized mice (Figure 4D; Figure S16, Table S2, Supporting Information). CD4^+^ T cell levels were considerably higher in NanoVac immunized mice, as compared with HA treated mice. Additionally, CD4^+^ T cell levels were increased even more after laser irradiation of mice immunized with NanoVac. Notably, CD8^+^ T cells, which are known as cytotoxic T cells, exhibited increased levels after immunization using NanoVac and irradiation with a laser (Figure 4E; Figure S16, Supporting Information). Increased levels of CD8^+^ T cells can effectively protect the host from viral infection and promote vaccine efficacy.^[^ ^71^, ^72^, ^73^ ^]^

To evaluate the activity of antibody‐productive B cells, plasma cells were detected by flow cytometry analysis using CD19 and CD138 markers in the bone marrow taken from the femurs of immunized mice (Figure 4F; Figure S17, Table S2, Supporting Information).^[^ ^74^, ^75^ ^]^ B cells triggered by antigens differentiate into plasma cells and produce antigen‐specific antibodies.^[^ ^76^ ^]^ Differentiated plasma cells do not express CD19, which is known as a hallmark of B cells, and are identified using CD138 instead. After immunization with NanoVac and irradiation, plasma cell levels were increased threefold relative to the case of free HA delivery, and by two‐fold relative to the case NanoVac immunization without laser irradiation.^[^ ^74^, ^76^ ^]^ Additionally, memory B cells were detected using CD19 and CD27 markers (Figure 4G; Figure S17, Supporting Information).^[^ ^74^, ^75^, ^77^ ^]^ After immunization with NanoVac and irradiation, memory B cell levels in treated mice were 2.3‐fold higher than that shown when only antigenic protein was delivered, and 1.4‐fold higher than that shown in the absence of laser irradiation.

These results indicate that vaccine delivery using the NanoVac formulation in combination with photochemical immunomodulation by laser irradiation induced maturation of DCs and helper T cells. Plasma cells and memory B cells were also elevated for enhancement of vaccine efficacy. Moreover, it was ascertained that NanoVac induced humoral immunity and cellular immunity, and further enhanced immune response via photochemical immunomodulation.

### 2.4 Antigen Specific Immune Response for Immunization with NanoVac and Laser Irradiation

**Figure 5:**
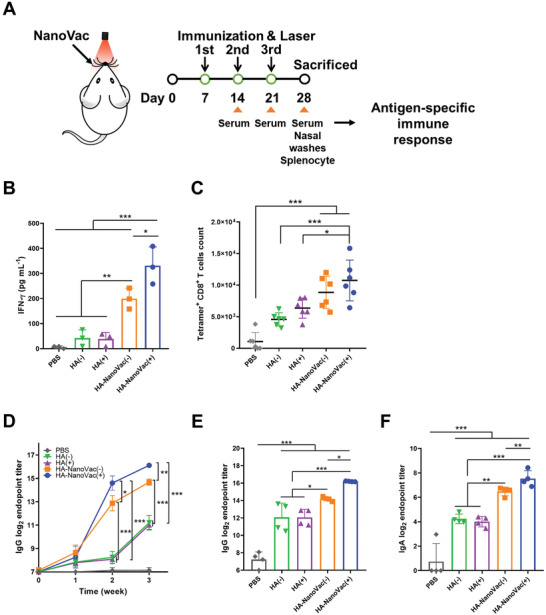
Increased of antigen‐specific immune response of immunization and laser irradiation with NanoVac. A) Schematic illustration of immunization and sample collection strategies. B) IFN‐*γ* cytokine levels were measured using ELISA from antigen (HA)‐boosted splenocytes of immunized mice (BALB/c, male, 6 weeks, *n* = 4). C) Antigen‐specific CD8^+^ T cells according to immunization and laser irradiation (Influenza HA Tetramer^+^ and CD3^+^/CD8^+^ cells in spleen, BALB/c, male, 6 weeks, *n* = 6). D) Confirmation of HA specific IgG antibody titer in serum during immunization schedules (three times immunization and laser irradiation, BALB/c, male 6 weeks, *n* = 3). E) HA‐specific IgG antibody titer in immunization endpoint. F) HA‐specific IgA antibody titer in nasal washes at immunization endpoint (BALB/c, male, 6 weeks, *n* = 4). Data expressed the means ± SD. Statistical significance was calculated using two‐way ANOVA and Dunnett's multiple comparison test by GraphPad Prism (* *P* < 0.05, ** *P* < 0.01, and *** *P* < 0.001).

To demonstrate the effectiveness of NanoVac for intranasal immunization, we explored the in vivo potential of NanoVac to determine whether it would effectively induce antigen‐specific responses (**Figure** **5A**).

To verify the HA‐specific immune response, production of the cytokine interferon‐gamma (IFN‐*γ*) was detected in splenocytes of immunized mice.^[^ ^61^, ^62^ ^]^ Splenocytes were isolated from the immunized mice treated with HA and HA‐NanoVac in the presence or absence of laser irradiation. The isolated splenocyte was boosted by HA protein, and then IFN‐*γ* secretion was evaluated. The secreted IFN‐*γ* level in the irradiated HA‐NanoVac‐immunized mice was 8.2‐fold higher than that in mice treated with free HA, and 1.7‐fold higher than that in mice immunized with HA‐NanoVac (Figure 5B).

The effectiveness of vaccination was confirmed by detecting the number of antigen‐specific CD8^+^ T cells. The number of HA‐specific CD8^+^ T cells was confirmed in the spleen of immunized mice using an influenza HA‐specific MHC tetramer. The number of HA‐specific CD8^+^ T cells in the mice immunized with NanoVac without laser irradiation was 1.6‐fold higher than only HA‐delivered mice group. After immunization with HA‐NanoVac and irradiation, the number of HA‐specific CD8

^+^ T cells was 2.0‐fold higher than those of free HA group and 1.2‐fold higher than those of NanoVac without laser irradiation group (Figure 5C; Figure S18, Supporting Information).

Serum immunoglobulin G (IgG) titer was measured using ELISA analysis to evaluate the HA‐specific immune response. HA‐specific serum IgG titer was increased to 27% in mice immunized with HA‐NanoVac (Figure 5D,E). Irradiation led to a further increase of 10% in the serum IgG titer, by virtue of temporally generated ROS which facilitates induction of immune response.^[^ ^40^, ^41^, ^42^, ^43^, ^44^, ^45^, ^46^, ^47^ ^]^ However, the serum IgG titer in free HA‐immunized mice was not affected, regardless of whether irradiation was applied.

To examine the contents of HA‐specific secreted IgA (SIgA) antibody response in nasal mucosa, the nasal washes were collected from immunized, and laser irradiated mice. Intranasal immunization focuses both on systemic immune responses and local immune responses.^[^ ^2^, ^3^, ^4^, ^24^ ^]^ In the absence of irradiation, the HA‐specific SIgA titer of the HA‐NanoVac‐immunized mice was more than 1.5‐fold higher than that of free HA‐immunized mice (Figure 5F). Moreover, laser irradiation of the HA‐NanoVac‐immunized mice resulted in an increase of 16% of the HA‐specific SIgA titer. Therefore, it was confirmed that the vaccine delivery system combined with NanoVac and laser irradiation elicited antigen‐specific immune response effectively.

Next, a hemagglutination inhibition (HAI) assay was performed to confirm that immunization with NanoVac and laser irradiation could protect against infectious influenza virus in vivo. After three times of immunization were completed, serum was collected from each group of mice. As a result, the highest HAI titer was elicited in the immunized mice by delivering HA‐NanoVac and laser irradiation (Figure S19, Supporting Information). Thus, we deduced that immunization with HA‐NanoVac and laser irradiation has a protective effect against the infectious influenza virus in the in vivo condition.

Additionally, the effective antigen delivery with the NanoVac formulation was further evaluated using OVA protein. The splenocytes of mice immunized with OVA and OVA‐NanoVac were isolated and boosted by OVA protein. The concentration of IFN‐*γ* secretion was measured using ELISA analysis (Figure S20A, Supporting Information). The secreted IFN‐*γ* concentration in the light‐irradiated OVA‐NanoVac‐immunized mice was threefold higher than that for mice treated with free OVA. In similar results shown in Figure 5, the highest titer of OVA‐specific IgG and SigA was detected in mice immunized with OVA‐NanoVac and irradiated with the laser (Figure S20B,C, Supporting Information). Immunohistochemical studies were performed for direct observation of SigA in the nasal cavity of mice (Figure S20D, Supporting Information). Confocal microscope images revealed that OVA‐NanoVac administration with additional laser irradiation increased SigA in the nasal cavity.

### 2.5 Validation of In Vivo Vaccine Efficacy with NanoVac

**Figure 6:**
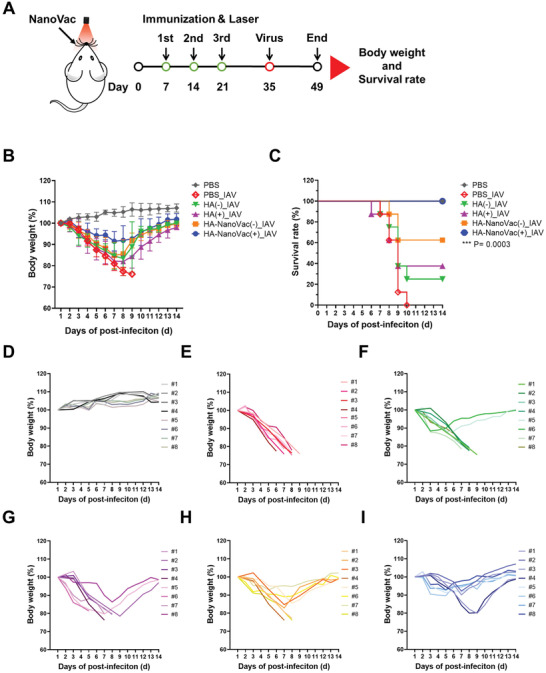
Vaccine efficacy of HA‐NanoVac against influenza A virus challenge. A) Schematic illustration of immunization schedules and virus challenge. Mice were inoculated with PBS, HA, and HA‐NanoVac in the presence (+) and absence (−) of laser irradiation (BALB/c male, 6 weeks, *n* = 8 mice per group). After 14 d of ended immunization, each mice group was challenged with influence A virus (15 LD50, 6 × 10^4^ pfu mouse^−1^). B) Body weight changes in 14 d after virus infection. C) Survival rate were monitored for 14 d. The statistical significance was indicated using the log‐rank (Mantel‐Cox) test (** *P* < 0.01, *P* = 0.0075). D–I) Body weight changes per mouse in different immunization group against influenza virus A infection. (D) PBS, (E) PBS_IAV, (F) HA(−)_IVA, (G) HA(+)_IAV, (H) HA‐NanoVac(−)_IAV, and (I) HA‐NanoVac(+)_IAV.

To evaluate whether immunization with HA‐NanoVac provides protection from a lethal infection with influenza virus (IAV, H1N1, A/California/07/09), mice immunized with HA‐NanoVac, free HA, PPA, and PBS were challenged with 15 times the 50% lethal dose (15 LD50, 6 × 10^4^ pfu mice^−1^, Figure S21, Supporting Information) of IAV (**Figure** **6A**). After virus infection, the mice were monitored for 14 days (d) to measure body weights and survival rate (Figure 6B,C; Figure S22, Table S1, Supporting Information). The body weight change as an indicator against infectious influenza virus challenges has been used to determine the effectiveness of the vaccine.^[^ ^78^, ^79^, ^80^ ^]^

The body weights of PBS‐ or free HA‐immunized mice without laser irradiation were drastically decreased and reached an experimental endpoint within 10 d after viral infection (Figure 6E,F). Immunization with free HA and laser irradiation did not provide protective immunity against viral infection. Among eight mice treated with both free HA and laser irradiation, five mice reached the experimental endpoint (Figure 6G). In immunization using HA‐NanoVac without laser irradiation, body weights decreased after virus infection, but gradually increased 7 d after infection and 62.5% of the mice survived the lethal challenge (Figure 6H). Remarkably, 100% of the mice immunized by a combination of HA‐NanoVac delivery and laser irradiation survived, and they exhibited 8.5% weight loss. In a group of mice immunized with HA‐NanoVac combined with laser irradiation, 75% of the mice showed a 6.4% decrease in their body weight 7 d after viral infection and the others had lost 20% of their initial body weight at a point 9 d after infection. Surprisingly, the body weights of all mice in this group gradually increased and they recovered completely (Figure 6I). Therefore, an immunization strategy combining NanoVac fabrication and laser irradiation served as an effective vaccine against lethal virus infection.

The histological hematoxylin and eosin (H&E) analysis further indicated that NanoVac and photochemical stimulation can be used as a safe delivery nanoplatform; these studies confirmed the absence of damage and toxicity in lung and nasal tissue after various sample administrations and laser irradiation (Figure S23, Supporting Information). In addition, OVA‐NanoVac was delivered, and histological analysis was performed. Histological damages were not observed in nasal cavities after OVA‐NanoVac was administered and laser irradiation applied (Figure S24, Supporting Information). These results showed that the NanoVac formulation can be used as a safe and effective vaccine with photochemical immunomodulation.

## 3 Conclusion

We demonstrate a novel and easy‐to‐fabricate nanoparticulate vaccine delivery platform, named NanoVac, composed of PPA and antigens with controlled photochemical immunomodulation. The NanoVac enables delivery of cargo antigens and prolongs residence times of the antigen protein in spite of mucociliary clearance mechanisms. Furthermore, a photochemical effect of the NanoVac formulation enhances the retention time of antigens through mucosal and tissue barriers without the use of permeation enhancers or mucolytic agents. Notably, an HA‐NanoVac consisting of influenza virus‐derived HA permits enhancement of DC maturation and HA‐specific immunity, including humoral and cellular immunity, and even enhances immunization efficacy via light‐triggered photochemical immunomodulation. Importantly, the combination of the NanoVac delivery system and a photochemical adjuvant effect demonstrates outstanding in vivo vaccine efficacy and successfully protects subject mice against pathogenic influenza virus infection. This new type of vaccine system can be used and engineered as a reliable protection against pathogenic virus infections, including those from influenza and COVID‐19.

## 4 Experimental Section

### Chemicals and Reagents

*β*‐Benzyl‐ʟ‐aspartate (Bz‐ʟ‐Asp), bis‐(trichloromethyl)‐carbonate (triphosgene), tetrahydrofuran (THF), *N*,*N*‐dimethylformamide (DMF), ethylenediamine (EDA), dimethyl sulfoxide (DMSO), deuterium oxide (D_2_O, 99.9 at%D), and H&E were purchased from Sigma‐Aldrich Co. (St. Louis, MO, USA). Dimethyl sulfoxide‐d6 (DMSO‐d_6_, 99.9 at%D; Cambridge Isotope Laboratories, Inc, Andover, USA). Chlorin e6 (Ce6; Frontier Scientific, Inc., Salt Lake City, UT, USA). Recombinant hemagglutinin of influenza virus (HA for H1N1, A/California/07/2009, Cat. No. 11085‐V08H) was purchased from Sino Biological, Inc. (Beijing, China). Horseradish peroxidase (HRP)‐conjugated goat antimouse antibodies (IgG and IgA) were purchased from Bethyl Laboratories, Inc. (Montgomery, TX, USA). Chicken red blood cells were purchased from Innovative Research Inc. (#ICHRBC5P15ML, MI, USA). The mouse IFN‐*γ* and IL‐2 enzyme‐linked immunosorbent assay kit were purchased from Enzo Life Science, Inc. (East Farmingdale, NY, USA). The mouse CCL3 (MIP‐1 *α*) and CCL4 (MIP‐1 *β*) ELISA kit were purchased from Abcam (Cambridge, UK). 3,3′,5,5′‐Tetramethylbenzidine (TMB) was purchased from SurModics, Inc. (Eden Prairie, MN, USA). Dialysis membranes (Spectra/Por; molecular weight cutoff size, MWCO 100–500 Da, 1 KDa, 3500 Da) were purchased from Spectrum Laboratories Inc. (Rancho Dominguez, CA, USA). DMEM medium, RPMI‐1640 medium, Dulbecco's phosphate buffered saline (DPBS), and penicillin/streptomycin solutions were purchased from HyClone (GE healthcare, IL, USA).

### Synthesis of *β*‐benzyl‐ʟ‐aspartic acid *N*‐carboxyanhydride (Bz‐ʟ‐Asp‐NCA)

Bz‐ʟ‐Asp‐NCA was synthesized according to a previously reported method.^[^ ^81^ ^]^ Briefly, Bz‐ʟ‐Asp (13.44 × 10^−3^ m, 3 g) was dissolved in THF (50 mL) containing triphosgene (10.11 × 10^−3^ m, 3 g) and stirred for 2 h at 60 °C. The crude mixture was filtered twice. The product was precipitated by hexane (900 mL) and recovered by filtration and drying under a vacuum at RT for 6 h to obtain Bz‐ʟ‐Asp‐NCA as a white powder.

### Synthesis of PBLA

PBLA was synthesized according to a previously reported method.^[^ ^81^ ^]^ First, PBLA was synthesized via ring‐opening polymerization of Bz‐ʟ‐Asp‐NCA initiated by the terminal amino group of butylamine. A solution of Bz‐ʟ‐Asp‐NCA (2.83 g, 11.36 mmol) in 20 mL DMF was added to butylamine solution (0.020 g, 0.24 mmol in 30 mL DMF). Then, the reaction mixture was stirred at RT for 2 d. PBLA was purified by precipitation in ether three times Vacuum dry afforded PBLA as a powder. The degree of polymerization of the Bz‐ʟ‐Asp units was calculated to be 37 from ^1^H‐NMR measurements. ^1^H‐NMR spectra were recorded at 300 MHz NMR spectrometer (Bruker, Germany) at 25 °C. ^1^H‐NMR (DMSO‐d_6_): *δ*0.80 (3H, C **H 3** CH_2_CH_2_CH_2_NH‐), *δ*2.82 (74H, —COCHC **H 2** COOCH_2_Ph), *δ*4.63 (37H, —COC **H** NH—), *δ*5.12 (74H, —COOC **H 2**—), *δ*7.34 (185H, —COOCH_2_ **Ph**), *δ*8.45 (37H, —COCHN **H**—).

### Synthesis of PBLA‐Ce6

Ce6 was conjugated to the terminal amine groups of PBLA via a conventional carbodiimide reaction. Separate solutions of PBLA (0.5 g, 0.061 mmol), a mixture of Ce6 (40 mg, 0.067 mmol), NHS (1.2 mol equivalent of Ce6), and DCC (1.2 mol equivalent of Ce6) in DMF (5 mL) were prepared. Then, the solutions were stirred vigorously for 3 h. Each of reactant solutions was mixed and stirred for 1 d at RT. Insoluble by‐products (e.g., dicyclohexylurea) were removed by filtration. Then, PBLA‐Ce6 was purified by precipitation in ether three times. Vacuum dry afforded PBLA as a powder. The production of PBLA‐Ce6 was verified by ^1^H‐NMR. ^1^H‐NMR spectra were recorded at 300 MHz NMR spectrometer. ^1^H‐NMR (DMSO‐d_6_): *δ*0.80 (3H, C **H 3** CH_2_CH_2_CH_2_NH—), *δ*2.82 (74H, —COCHC **H 2** COOCH_2_Ph), *δ*4.63 (37H, —COC **H** NH—), *δ*5.12 (74H, —COOC **H 2**—), *δ*7.34 (185H, —COOCH_2_ **Ph**), *δ*8.45 (37H, —COCHN **H**—), *δ*9.15 (1H, **20‐H** of Ce6), *δ*9.75‐9.80 (2H, **5‐ and 10‐H** of Ce6).

### Synthesis of Poly[(2‐aminoethyl)aspartamide]‐chlorin e6 (Photoactivatable Polymeric Adjuvant)

EDA (2.30 × 10^−3^ m) was slowly added to the PBLA‐Ce6 solution (0.2 g, 0.023 × 10^−3^ m in 10 mL DMSO), and the reactant solution was stirred for 6 h under a nitrogen atmosphere. The resulting solution was dialyzed three times against ice‐cold 10 × 10^−3^ m HCl and distilled water. After lyophilization, PPA was obtained as a powder. The actual content of (2‐aminoethyl)aspartamide was determined to be 29 (79%) of total aspartic acid units (37), and eight (21%) of aspartic acid remained from ^1^H‐NMR measurements. ^1^H‐NMR spectra were recorded at 300 MHz NMR spectrometer. ^1^H‐NMR (D_2_O):*δ*0.85 (6H, C **H 3** CH_2_CH_2_CH_2_NH— and **18 ′ ‐H** of Ce6), *δ*1.25 (4H, CH_3_C **H 2** C **H 2** CH_2_NH—) *δ*1.86 (9H, **2 ′ ‐, 7 ′ ‐ , and 12 ′ ‐H** of Ce6), *δ*2.50‐2.95 (74H, —COCHC **H 2** COOCH_2_Ph), *δ*3.00‐3.20 (58H, —CONHCH_2_C **H 2** NH_2_), *δ*3.35‐3.55 (58H, —CONHC **H 2** CH_2_NH_2_), *δ*4.54‐4.74 (37H, —COC **H** NH—).

### Gel Permeation Chromatography

Samples were analyzed using high performance liquid chromatography with a GPC KF‐804 and GPC KF‐805 column (Shodex, Tokyo, Japan) with *N*,*N*‐dimethylformamide as the mobile phase at a flow rate of 1.0 mL min^−1^. Narrow molecular weight poly(ethylene glycol) standards were used to calibrate the average molecular weights of samples.

### Preparation of NanoVac

NanoVac was formulated according to a previously reported method by a simple mixing process.^[^ ^82^ ^]^ Briefly, HA (distilled water, DW; 0.050 g L^−1^) was added to different concentrations (DW, 0.025, 0.050, 0.250, 0.500, and 1000 g L^−1^) of PPA solution with an equivalent volume at RT. The formulation condition of the polymer/antigen complex was investigated based on the weight ratio of polymer to antigen. When performing the in vitro and in vivo experiments, HA and PPA were dissolved in PBS (pH 7.4) and used.

### Characterization of NanoVac

The morphology of NanoVacs was observed under field emission scanning electron microscopy (S‐4800; Hitachi, Japan). The hydrodynamic diameters and particle size distributions of NanoVacs and their zeta potentials were measured on a DLS analyzer using an electrophoretic light scattering photometer (Zetasizer Nano ZS, Malvern Instruments Ltd., UK). NavoVac dispersion was passed through syringe filters (0.45 µm, Millipore) before measurements. DLS was performed at 25 °C in DW.

The absorbance spectra of Ce6 and PPA were measured using UV–vis spectrophotometer (UV‐2700; Shimadzu, Japan). The Ce6 content of the PPA conjugates was analyzed at 663 nm. Free Ce6 and PPA were dissolved in 4:1 (v/v) DMSO/D.I. water in 0.042 × 10^−3^ m Ce6 concentration. Comparison of absorption spectra among Ce6, PPA, and HA‐NanoVac was performed in D.I. water (0.042 × 10^−3^ m Ce6).

Fluorescence intensities of Ce6, PPA, and HA‐NanoVac were measured at 662 nm using spectrofluorophotometer (RF‐5301PC; Shimadzu, Japan). Ce6, PPA, and HA‐NanoVac were dissolved in D.I. water in 0.042 × 10^−3^ m Ce6 concentration.

Singlet oxygen generation was compared with Ce6, PPA, and HA‐NanoVac. Singlet oxygen was detected by SOSG as a probe. Ce6, PPA, and HA‐NanoVac were dissolved in D.I. water and added with 2 × 10^−3^ m SOSG solution. Each mixed solution was exposed with laser until 20 J cm^−2^ (671 nm, LaserLab, Korea). The fluorescence intensity of SOSG was measured by spectrofluorophotometer (Ex, 494 nm; Em, 534 nm).

### Time‐Resolved Singlet‐Oxygen Production Measurements

Singlet‐oxygen was analyzed by direct detection of near‐IR luminescence emission of oxygen at 1270 nm.^[^ ^83^ ^]^ The samples were excited with 671 nm µJ pulses (5 µs duration) produced by a fiber‐coupled laser performing with a 10 kHz repetition rate. The singlet‐oxygen luminescence was measured by a photomultiplier tube (PMT) detector (H10330‐45, Hamamatsu, Shizuoka, Japan). Three bandpass filters (1220, 1270, and 1320 nm) were located orderly to fore position of the photodetector to sample the luminescence spectrum.

### Circular Dichroism Spectroscopy

HA protein and HA‐NanoVac were prepared in PBS (pH 7.4) and DW to a concentration of 0.2 mg mL^−1^ HA protein (1.0 mg mL^−1^ PPA in HA‐NanoVac). Each sample was measured in presence (+) or absence of laser irradiation (671 nm, 1 J cm^−2^). A spectrum was measured at 25 °C between 195 and 250 nm using 1 mm path length, with a 1 nm bandwidth. CD experiments were performed using CD spectrophotometer (J‐815, Jasco, Japan). The three equivalent spectra of each sample were averaged, and baseline determined using PBS buffer or DW alone.

### SDS‐PAGE and Western Blot

HA protein (H1N1, A/California/07/2009, Sino Biological) and HA‐NanoVacs denatured at 95 °C and loaded onto 12% polyacrylamide gel containing (SDS. Gel images were obtained using ChemiDoc MP gel imaging system (Bio‐Rad, USA). After SDS‐PAGE, the gel was transferred to PVDF membrane (Bio‐Rad, USA). The membrane was blocked using 5% skim milk in TBST (tris‐buffered saline containing 0.1% tween 20, v/v) for 1 h. HA proteins were detected using anti‐HA antibodies (Santa Cruz Biotechnology, sc‐52025). These primary antibodies were detected by HRP‐conjugated secondary antibodies (Bethyl Laboratories, A90‐116P). Blotting was performed using ChemiDoc MP gel imaging system.

### Cell Culture

Madin‐Darby Canine Kidney (MDCK) cells, WI38 cells, L929 cells, and RPMI‐2650 cells were obtained from the Korean Cell line Bank (KCLB). MDCK cells (KCLB; #10034) were cultured in DMEM (HyClone, GE healthcare, USA) containing 10% FBS (HyClone, USA) and 1% antibiotics‐antimycotics. WI 38 cells (KCLB; #10075) were maintained in EMEM (ATCC, USA) including 10% FBS and 1% antibiotics‐antimycotics. L929 cells (KCLB; #10001) and RPMI‐2650 cells (KCLB; #10030) were maintained in RPMI‐1640 medium (HyClone, USA) including 10% FBS and 1% antibiotics‐antimycotics. All cells were cultured at 37 °C and 5% CO_2_ in a humidified incubator.

### In Vitro Cytotoxicity of PPA and NanoVac

To evaluate the cytotoxicity of PPA and NanoVac, MDCK cells, WI38 cells, L929 cells, and RPMI‐2650 cells were seeded at 5 × 10^3^ cell per well in 96‐well plate. PPA and NanoVac was diluted to various concentrations in serum free medium and incubated with each cell line for 4 h at 37 °C and 5% CO_2_. Additionally, cells were irradiated with laser to confirm phototoxicity of PPA and NanoVac (671 nm, 1 J cm^−2^). After 4 h postincubation, cells were washed twice with PBS. Cell viability was analyzed via MTT assay. To detect the generated formazan, each plate was dissolved in DMSO and transferred to a new plate. Absorption intensity was measured at 571 nm using microplate reader (Bio‐Tek, USA).

### Animal Experiments

All procedures were approved by the Institutional Animal Care and Use Committee (IACUC) of the Catholic University of Korea. Experiment was executed following the guideline of the “WHO Manual on Animal Influenza Diagnosis and Surveillance, 2002,” “Manual for the laboratory diagnosis and virological surveillance of influenza, 2012” by World Health Organization and “Guide for the Care and Use of Laboratory Animals, 8th edition, 2011” by National Research Council (USA). Male BALB/c and BALB/c nude mice aged 6 weeks (Orient Bio, Inc., Republic of Korea) were used, and cages were ventilated under controlled temperature (24 ± 1 °C) and humidity (60% ± 5%) conditions. Mice were anesthetized by isoflurane inhalation. Animal were euthanized by CO_2_ gas when reached experimental end point.

### Fluorescence Image of Mouse Nasal Region

To detect the HA protein in the nose of mouse, HA protein was labeled by FITC. NanoVac was formulated using F‐HA and PPA in the same condition as described above (F‐HA‐NanoVac). PBS, F‐HA, and F‐HA‐NanoVac were administered intranasally to mice (BALB/c nude, male, 6 weeks, *n* = 4), and fluorescence images were observed for 24 h. Green fluorescence was measured using FOBI (Neo Science, Korea).

### Fluorescence Image of Sectional Nasal Region in Time Courses

The areas of the nasal tissues were excised after the administration at various time (1, 3, 6, 12, 24 h), perfused in 4% paraformaldehyde, dehydrated in 10%, 20%, and 30% sucrose solutions, and then frozen sectioned for detection of F‐HA. Frozen sections from sample administered mice were subjected to a blocking step with 1% BSA, stained with DAPI, and analyzed using confocal microscopy (LSM 710 Meta, Carl Zeiss, Germany). The nasal septum regions were selected using Fiji/ImageJ, and mean fluorescence intensity of sectional nasal tissue were analyzed.

### In Vitro BMDCs Maturation

Bone marrow‐derived monocytes were isolated from removed femurs and tibias and the marrow flushed with PBS. Isolated monocytes were cultured in RPMI 1640 media containing IL‐4 (10 ng mL^−1^_,_ BioLegend, USA) and GM‐CSF (20 ng mL^−1^_,_ BioLegend, USA) for 6 d to induce immature DCs. In vitro cultured BMDCs were seeded at 2 × 10^5^ cells per well in 12‐well plate and incubated for 24 h. After 24 h, cultured immature DCs were treated with various sample groups such as PBS, PPA, HA, and HA‐NanoVac (HA 2 µg mL^−1^, PPA 10 µg mL^−1^). After sample treatment, inoculated cells were compared in the presence or absence of laser irradiation (1 J cm^−2^). DCs maturation was detected using immunostaining with antibodies against CD11c (FITC‐conjugated), CD86 (APC‐conjugated), and CD80 (PE‐conjugated) after Fc receptor blocking procedure at 4 °C for 30 min. To identify dendritic cells and maturation, flow cytometry was conducted and analyzed using an FACS Canto II (BD Biosciences, USA). FlowJo software version 10 was used for data analysis.

### Immunization and Sample Collection

BALB/c mice (*n* = 5) were immunized intranasally using free HA and HA‐NanoVac (10 µg of HA and 50 µg of PPA) dissolved in 20 µL PBS. Half an hour later, nose regions of immunized mice were illuminated by a 671 nm fiber‐coupled diode laser system (50 J cm^−2^, LaserLab, Republic of Korea). The administration and light irradiation were repeatedly performed three times at 1‐week intervals. All mice were anesthetized before the next immunization. Blood was collected from immunized mice via the retro orbital plexus and submandibular one week after each vaccination and incubated at RT for 30 min. The sera were collected from the blood by centrifugation (13 000 rpm, 20 min). Nasal washes were collected by flushing with 200 µL of PBS through the nasal cavity from sacrificed mice. All serum and nasal washes were stored at −78 °C prior to use.

### Determination of BMDCs Maturation of Immunized Mice

Femurs and tibias of immunized mice were dissected 7 days after immunization. Bone marrow‐derived monocytes were isolated from removed femurs and tibias and the marrow flushed with PBS. Isolated monocytes were cultured in RPMI 1640 media containing IL‐4 (10 ng mL^−1^, BioLegend, USA) and GM‐CSF (20 ng mL^−1^_,_ BioLegend, USA) for 24 h. DCs and maturation were confirmed using immunostaining with antibodies against CD11c (FITC‐conjugated), CD86 (APC‐conjugated), and CD80 (PE‐conjugated) after Fc receptor blocking procedure at 4 °C for 30 min. To identify dendritic cells and maturation, flow cytometry was conducted and analyzed using an FACS Canto II (BD Biosciences, USA). FlowJo software version 10 was used for data analysis.

### Determination of Dendritic Cell Maturation

The lymph nodes of mice were dissected from immunized and laser irradiated mice (three times; PBS, PPA(−), HA(−), HA‐NanoVac(−), PPA(+), HA(+), and HA‐NanoVac(+); BALB/c male, 6 weeks, *n* = 4). Harvested lymph nodes were homogenized and treated with red blood cell (RBC) lysis buffer (Sigma‐Aldrich, MO, USA) briefly, then centrifuged at 1500 rpm for 3 min to obtain cells. DCs and maturation were confirmed using immunostaining with antibodies against CD11c (FITC‐conjugated), CD86 (APC‐conjugated), and CD80 (PE‐conjugated) after Fc receptor blocking procedure at 4 °C for 30 min. To identify dendritic cells and maturation, flow cytometry was conducted and analyzed using an FACS Canto II (BD Biosciences, USA). FlowJo software version 10 was used for data analysis.

### Cell Population of T Cell and B Cell in Immunized Mouse

To confirm the amount of T cells and B cells according to immunization, spleen and femur were dissected from three times immunized and laser irradiated mice (BALB/c, male, 6 weeks, *n* = 3 for each analysis). Spleens were harvested from immunized mice for determination of the T cell population. It was homogenized and treated with RBC lysis buffer briefly, then centrifuged to obtain cells (1500 rpm, 3 min). To identify CD4 helper T cells and CD8 cytotoxic T cells, T cells were gated as CD3^+^ before CD4^+^ and CD8^+^ were applied. T cells were detected using immunostaining with antibodies against CD3 (FITC‐conjugated), CD4 (APC‐conjugated) and CD8 (PE‐conjugated) at 4 °C for 30 min after Fc receptor blocking procedure.

Bone marrow was obtained from immunized mice for determination of the B cell population. Mouse femur was flushed to collect the contents of marrow using syringe and cells were collected with PBS. To identify plasma B cells and memory B cells, B cells were detected with antibodies against CD19 (FITC‐conjugated), CD27 (APC‐conjugated), and CD138 (PE‐conjugated) at 4 °C for 30 min after Fc receptor blocking procedure. Flow cytometry analysis was executed using an FACS Canto II (BD Biosciences, USA). FlowJo software version 10 was used for data analysis.

### Antigen Specific Antibody Detection

Antigen (HA or OVA)‐specific IgG and IgA antibody titers were determined by ELISA kit using the serum or nasal washes from each mouse (*n* = 5). Briefly, 96‐well ELISA plates were precoated with 100 µL of HA (1 µg mL^−1^, or OVA 1 µg mL^−1^) overnight at 4 °C. After blocking with PBS containing 2% BSA for 1 h at RT, twofold diluted sample solutions (100 µL in PBS containing 2% BSA) were added to each well. Then, 1:20 000 or 1:10 000 diluted HRP‐conjugated antimouse antibodies were added (IgG or IgA). After incubation (1 h at RT), TMB (100 µL) was added to each well. The reaction termination solution of 2 N sulfuric acid was added. The absorbance was recorded by a multiplate reader at a wavelength of 450 nm (Bio‐Tek, VT, USA). The endpoint titer was decided by an O.D. cut‐off value of 0.2.

### Detection of IFN‐*γ* Cytokine in Splenocyte

Spleens from immunized mice were dissected and cell suspensions were obtained. Cells were washed once with RPMI 1640 containing 10% fetal calf serum (heat‐inactivated, endotoxin‐free; FCS, Gibco), streptomycin (100 mg mL^−1^), and penicillin (100 U mL^−1^). RBCs were removed by RBC lysis buffer and the remaining cells were washed twice in cold DPBS. Cytokine generation was assessed by culturing splenocytes (10^7^ cells mL^−1^) in triplicate with HA (10 mg mL^−1^ or OVA 10 mg mL^−1^). Control stimuli included RPMI 1640 medium only. Supernatants were harvested after 72 h at 37 °C, 5% CO_2_ condition, filtered and stored at −78 °C before use. For quantification of the Th1‐associated cytokine IFN‐*γ*, ELISA was used following the manufacturer's protocols. Briefly, 50 µL of twofold diluted samples in the blocking buffer were added into the precoated mouse IFN‐*γ* Microtiter 96‐well plate and incubated with biotinylated IFN‐*γ* antibody for 2 h at RT. Then, HRP–streptavidin conjugates (100 µL well^−1^) were added and incubated for 30 min. The reaction termination of 0.18 m sulfuric acid was added. The absorbance was recorded by a microplate reader at 450 nm (Bio‐Tek, USA). The IFN‐*γ* production of the sample was calibrated against recombinant mouse IFN‐*γ* standards.

### Antigen‐Specific CD8 T Cell Population in Immunized Mouse

To confirm the amount of antigen‐specific CD8^+^ T cells according to immunization, spleen was dissected from three times immunized and laser irradiated mice (BALB/c, male, 6 weeks, *n* = 6 for each analysis). Spleens were harvested from immunized mice for the determination of T cell population. It was homogenized and treated with RBC lysis buffer briefly, then centrifuged to obtain cells (1500 rpm, 3 min). To identify antigen‐specific CD8^+^ T cells, T cells were detected using immunostaining with antibodies (CD3‐FITC, CD8‐PE) and MHC tetramer (Influenza HA Tetramer APC‐conjugated, #TS‐M535‐2, MBL Science Co. LTD., Japan) at 4 °C for 30 min after Fc receptor blocking procedure. Flow cytometry analysis was executed using an FACS Canto II (BD Biosciences, USA). FlowJo software version 10 was used for data analysis.

### Detection of Cytokines in Sera

Blood was collected from submandibular area of immunized mice. To obtain serum, collected blood was centrifuged at 13 000 rpm for 20 min. Supernatants were obtained after centrifugation and stored at −78 °C before use. IL‐2 (ADI‐900‐042), CCL3 (ab200017), and CCL4 (ab215115) were detected by ELISA that according to the manufacturer's protocol.

### Virus

Influenza A/California/07/2009 (H1N1) virus was used for the experiment. It was obtained from the Korea Disease Control and Prevention Agency. The virus was cultured in MDCK cells in the TPCK treated DMEM‐BSA for 3–5 d at 37 °C, 5% CO_2_.^[^ ^84^ ^]^ Plaque titration of influenza A virus was performed on MDCK cells in the presence of 0.25% trypsin at 37 °C, 5% CO_2_. The yield of the virus is about 2 × 10^7^ plaque‐forming units (pfu) mL^−1^.

### Virus Isolations

Virus cultivation medium (TPCK treated DMEM‐BSA) was harvested and centrifuged to remove the cell debris (3200 × *g* at 4 °C for 10 min). The collected supernatant was subjected to ultracentrifugation (112 000 × *g*, 4 °C for 1.5 h, Hitachi CP‐100WX, P28S rotor) with 10% Optiprep cushioning. The supernatant was discarded, and the pellet virions were suspended in 1 mL 1X NTC buffer (100 × 10^−3^ m NaCl, 20 × 10^−3^ m Tris‐HCl pH 7.4, 5 × 10^−3^ m CaCl_2_). The suspended virions were loaded onto a discontinuous Optiprep gradient (10%, 15%, 20%, 25%, and 30% in NTC buffer), which was subjected to ultracentrifugation in a P90AT rotor at 4 °C for 2.5 h at 175 000 × *g*.

Afterward, the virions existed as a visible white band in the 25%–30% gradient section. After ultracentrifugation, the virions were extracted including the visible white band. The extracted virions were concentrated by ultracentrifugation (180 000 × *g*, 4 °C for 2.5 h, P90AT rotor) in ice‐cold NTC buffer. The pellet was suspended in ice‐cold NTC buffer and the aliquots were retained and stored at −78 °C.^[^ ^84^, ^85^ ^]^

### Hemagglutination Inhibition Assay

Serum was collected after three times immunized and laser irradiated mice (BALB/c, male, 6 weeks, *n* = 8). These sera were treated with receptor destroying enzyme from *Vibrio Cholerae* (Denka Seiken, Japan) and incubated overnight at 37 °C. After incubation, the samples were heat‐inactivated at 56 °C for 30 min. The serum samples were diluted at 1:10 from an initial. The diluted sera were added with 4‐HA units and incubated for 30 min at room temperature. After 30 min 0.75% of chicken red blood cells were added and incubated for 30 min at room temperature. The limit of detection of this assay is 1:10. When the titer was below 1:10, the results were considered negative and expressed as 5 for the calculation of geometric mean titers.

### Determination of the 50% Lethal Dose (LD50) of Influenza A/California/07/2009 (H1N1) Virus in Mice

Mice (BALB/c male, 6 weeks, *n* = 8) were infected intranasally with different doses of influenza virus (3 × 10^3^ to 3 × 10^5^ pfu mouse^−1^ in 50 µL of PBS). After infection, the percent changes in body weight and survival were monitored for 14 d. The LD50 of influenza virus in mice was determined by the Reed–Muench method.^[^ ^86^ ^]^ The experimental endpoint was determined at a point when the body weight was reduced to less than 25% or death. Euthanasia of mice was carried out at the survival endpoint.

### Influenza Virus Challenge

BALB/c mice (6 weeks, male, *n* = 8 mice per group) were immunized intranasally with PPA, free HA, and HA‐NanoVac (10 µg of HA, 50 µg of PPA) in 20 µL of PBS. PBS‐treated mice were used as a negative control. Within 30 min after sample administration, nose regions of immunized mice were irradiated with laser (671 nm, 50 J cm^−2^). The administration and laser irradiation were repeated three times at 1‐week intervals. Non‐irradiated mice were compared with laser exposed group. Two weeks after vaccination was over, immunized mice were challenged with influenza A virus (A/California/07/2009, H1N1). After infection with virus (15 LD50, 6 × 10^4^ pfu mouse^−1^), the body weight change and survival were monitored for 14 d. The survival endpoint was determined at a point when the body weight was reduced to less than 25% compared with the initial body weight or death. Euthanasia of mice was carried out at the survival endpoint.

### Immunohistochemical and Histological Analysis

The areas around the nasal tissues were excised at 7 d after the immunization, perfused in 4% paraformaldehyde, dehydrated in 10%, 20%, and 30% sucrose solutions, and then frozen sectioned (20 µm) for FITC‐conjugated antimouse IgA (BD Biosciences) counter staining and H&E staining. Frozen sections from immunized mice were subjected to a blocking step with 1% BSA and were stained with FITC‐conjugated antimouse IgA antibody. The sections were then counterstained with DAPI and analyzed using confocal microscopy (LSM 710 Meta, Carl Zeiss, Germany). For H&E staining, the sectioned nasal tissues were stained with H&E for the nucleus and cytoplasm. The stained sections were captured using slide scanner (APERIO CS2, Leica Biosystems, Germany).

### Statistical Analysis

Data are expressed as the mean ± standard deviation (SD). Differences between the values were assessed using Student's *t*‐test and two‐way ANOVA via Tukey and Dunnett's multiple compared tests. Mouse survival rate were determined using the log‐rank (Mantel‐Cox) test. All analyses for statistically significant differences were calculated via GraphPad Prism 8.0, with significance being indicated by *p* values of <0.001 (***), <0.01 (**), and <0.05 (*).

## Conflict of Interest

The authors declare no conflict of interest.

## Supporting information

Supporting Information

Supporting Information

## Data Availability

Research data are not shared.
