# Supplementary material for: Hemagglutinin Nanoparticulate Vaccine with Controlled Photochemical Immunomodulation for Pathogenic Influenza‐Specific Immunity
Source: Adv Sci (Weinh). 2021 Oct 24;8(23):2100118. doi: 10.1002/advs.202100118 (PMC8655185; doi:10.1002/advs.202100118)
Supplement: Supplementary file 1 — Supporting Information [file ADVS-8-2100118-s001.pdf]

ADVANCED SCIENCE
Open Access

Supporting Information

for *Adv. Sci.*, DOI: 10.1002/advs.202100118

Hemagglutinin nanoparticulate vaccine with controlled photochemical immunomodulation for pathogenic influenza-specific immunity

*Hayoon Jeong, Chung-Sung Lee, Jangsu Lee, Jonghwan Lee, Hee Sook Hwang, Min Lee and Kun Na**

Supporting Information

Hemagglutinin nanoparticulate vaccine with controlled photochemical immunomodulation for pathogenic influenza-specific immunity

*Hayoon Jeong, Chung-Sung Lee, Jangsu Lee, Jonghwan Lee, Hee Sook Hwang, Min Lee and Kun Na**

H. Jeong, J. Lee and Prof. K. Na
Department of Biomedical-Chemical Engineering,
The Catholic University of Korea,
Bucheon-si, Gyeonggi-do, 14662, Republic of Korea
E-mail: kna6997@catholic.ac.kr

H. Jeong, Prof. C. S. Lee, J. Lee, Dr. J. Lee, Prof. H. S. Hwang and Prof. K. Na
Department of Biotechnology,
The Catholic University of Korea,
Bucheon-si, Gyeonggi-do, 14662, Republic of Korea
E-mail: kna6997@catholic.ac.kr

Prof. C. S. Lee and Prof. M. Lee
Division of Advanced Prosthodontics,
University of California Los Angeles, Los Angeles, CA, 90095, USA

Prof. C. S. Lee
Department of Pharmaceutical Engineering and Biotechnology,
Sun Moon University,
Asan-si, Chungcheongnam-do 31460, Republic of Korea

Prof. H. S. Hwang
Department of Pharmaceutical Engineering,
Dankook University,
Cheonan-si, Chungcheongnam-do 31116, Republic of Korea

Prof. M. Lee
Department of Bioengineering,
University of California Los Angeles, Los Angeles, CA, 90095, USA

Hayoon Jeong, Chung-Sung Lee and Jangsu Lee contributed equally.

*Corresponding author: Kun Na, Ph.D.

Department of Biomedical-Chemical Engineering, Department of Biotechnology,

The Catholic University of Korea,

43 Jibong-ro, Bucheon-si, Gyeonggi-do, 14662, Korea

Tel: +82-2-2164-4832

Fax: +82-2-2164-4865;

E-mail: kna6997@catholic.ac.kr

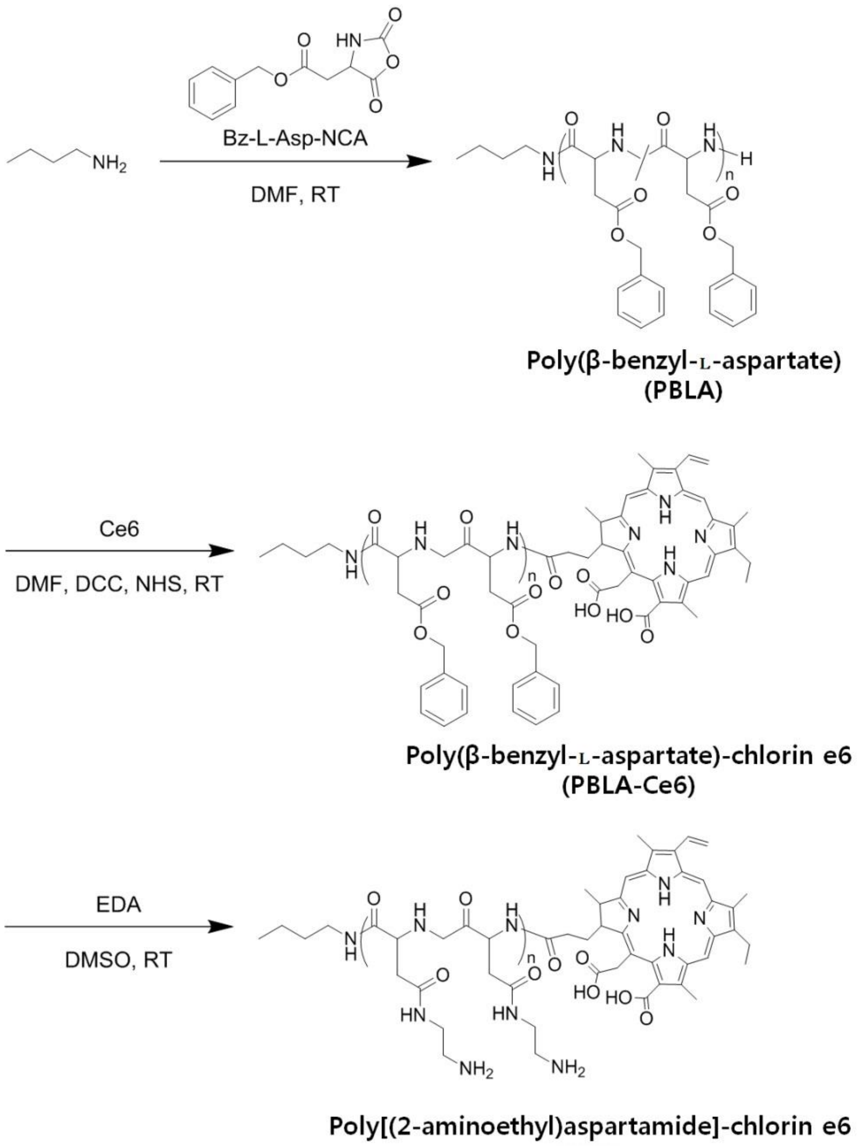

Figure S1. Synthetic route of photoactivatable polymeric adjuvant (PPA), poly[(2-aminoethyl)aspartamide]-chlorin e6.

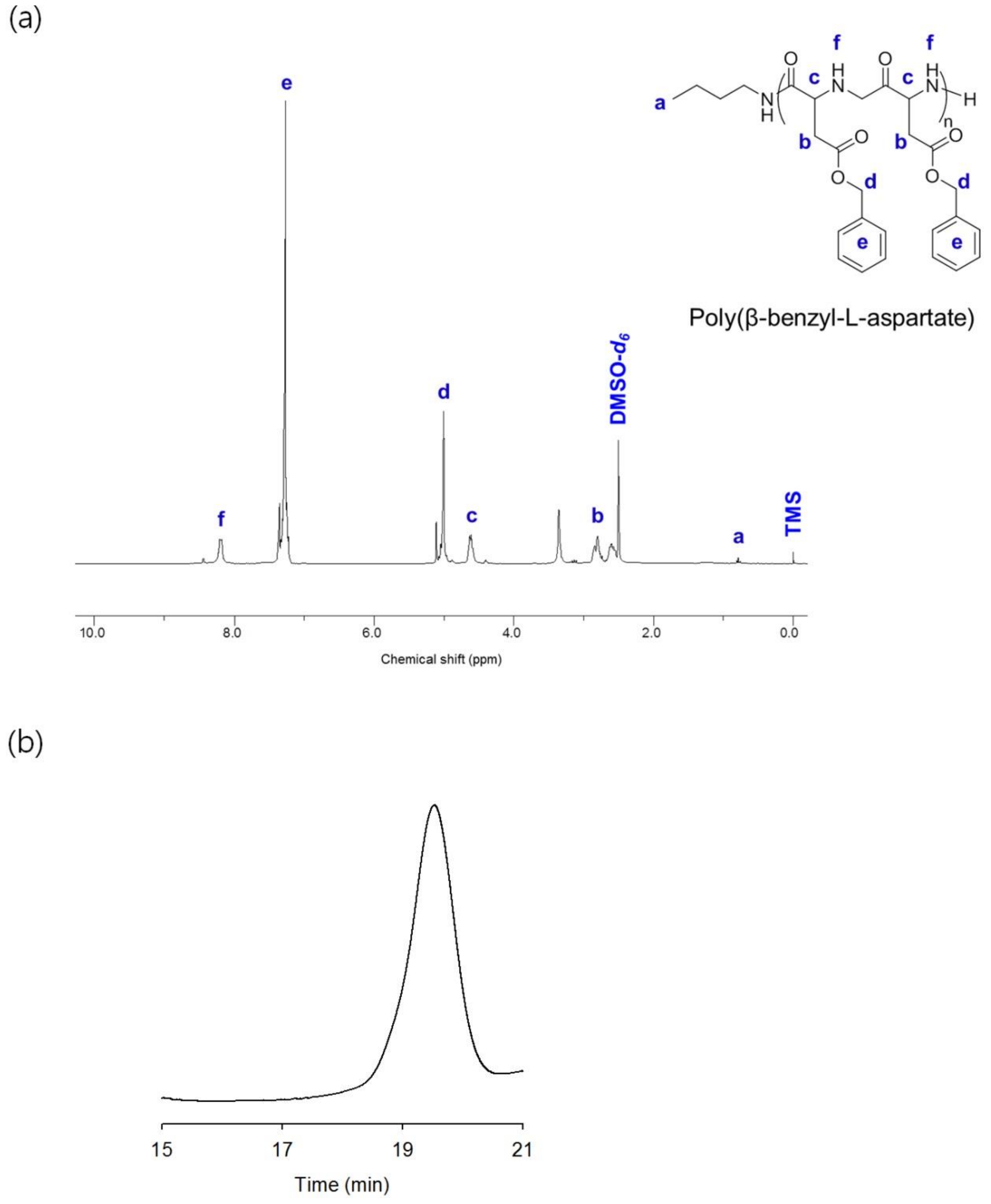

Figure S2. a) ^{1}H-NMR analysis of poly(β-benzyl-L-aspartate) (PBLA) in DMSO-d_6. The degree of polymerization (DP) of the Bz-L-Asp units was calculated to be 37 from ^{1}H-NMR measurements. b) GPC curve of PBLA. (Mw: 12,480, Mn: 7,900, PDI: 1.58)

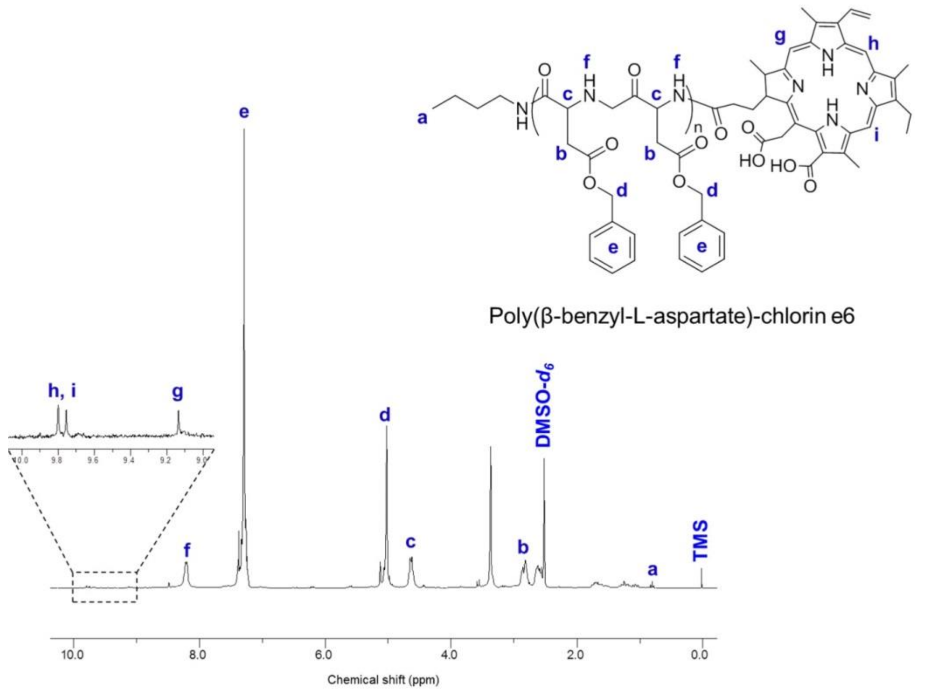

Figure S3. ^{1}H-NMR analysis of poly(β-benzyl-L-aspartate)-chlorin e6 (PBLA-Ce6) in DMSO-d_6.

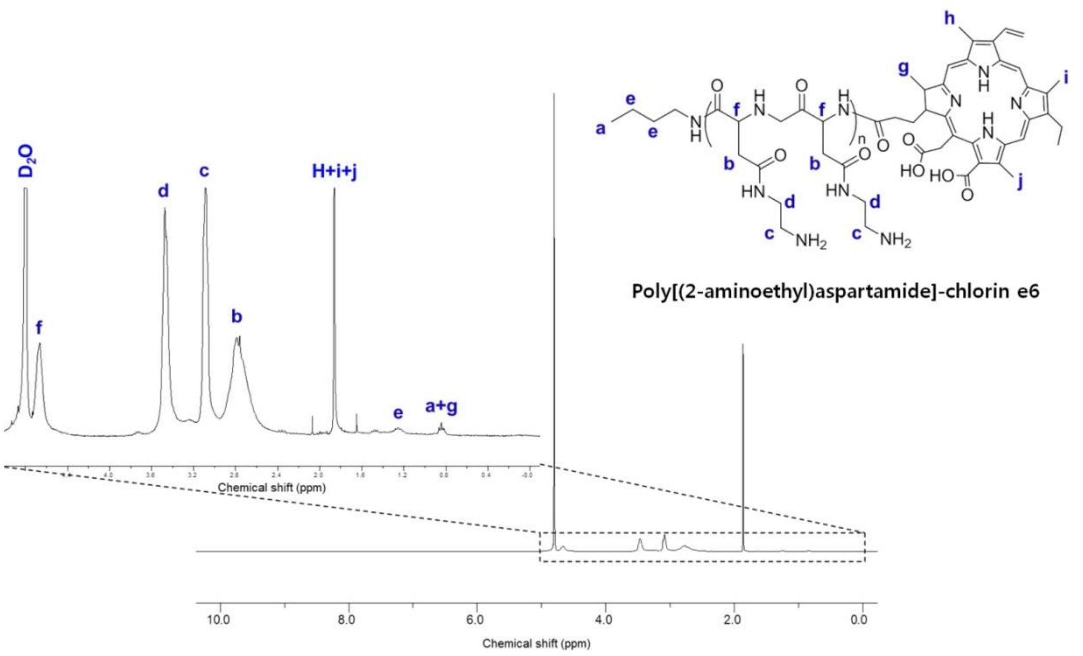

Figure S4. ^{1}H-NMR analysis of poly[(2-aminoethyl)aspartamide]-chlorin e6 (Photoactivatable polymeric adjuvant, PPA) in D_2O.

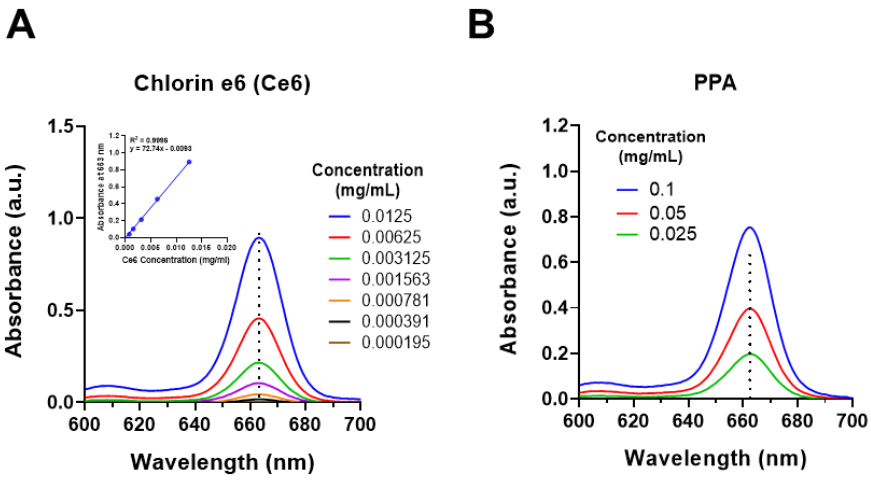

Figure S5. UV-Vis absorbance and fluorescence intensity of PPA. A) UV-Vis spectra of Chlorin e6 were measured in various concentrations. A standard curve was calculated according to absorbance at 663 nm. B) UV-Vis spectra of PPA were measured in different concentrations. Ce6 and PPA were dissolved in DMSO:D.I. water solutions (DMSO:D.I water = 4:1) for UV-vis absorbance spectrum analysis.

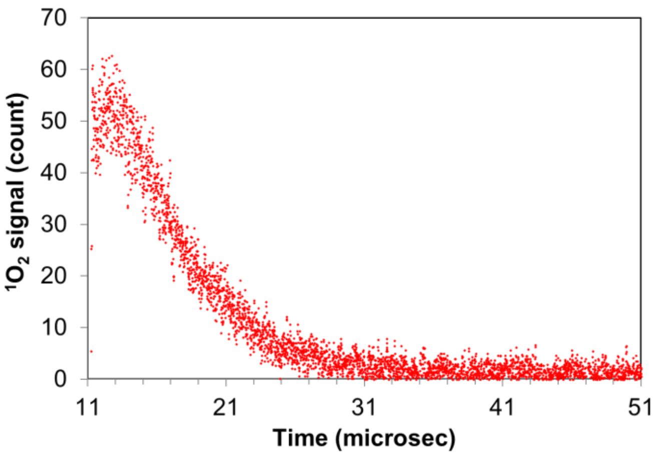

Figure S6. Photo-mediated activation of PPAs. Kinetics of singlet-oxygen luminescence of PPAs under laser irradiation of 670 nm μJ pulses (5 μs duration) generated by a fiber-coupled diode laser operating with 10 kHz repetition rate.

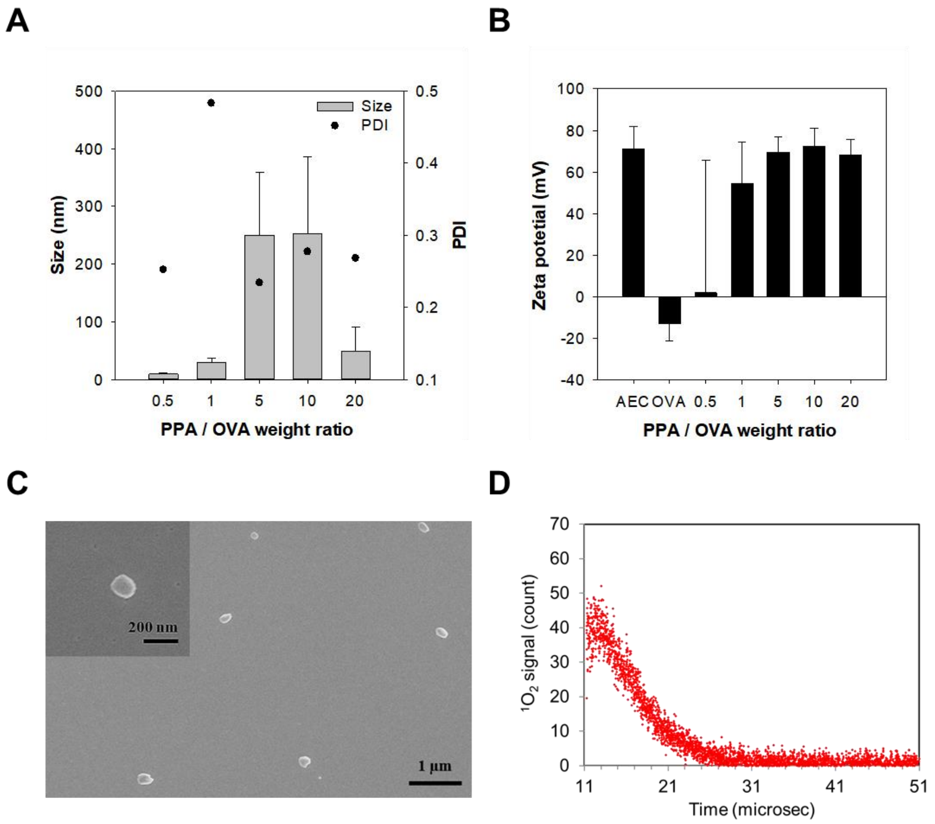

Figure S7. Characterization of OVA-NanoVacs. The effect of PPA/OVA weight ratios on A) size, PDI value and B) ζ-potential of OVA-NanoVacs. C) Field emission-scanning electron microscopy (FE-SEM) image of OVA-NanoVacs at a PPA/OVA weight ratio of 5. D) Photo-mediated activation of OVA-NanoVacs. Kinetics of singlet-oxygen luminescence of OVA-NanoVacs under laser irradiation of 670 nm μJ pulses (5 μs duration) generated by a fiber-coupled diode laser operating with 10 kHz repetition rate.

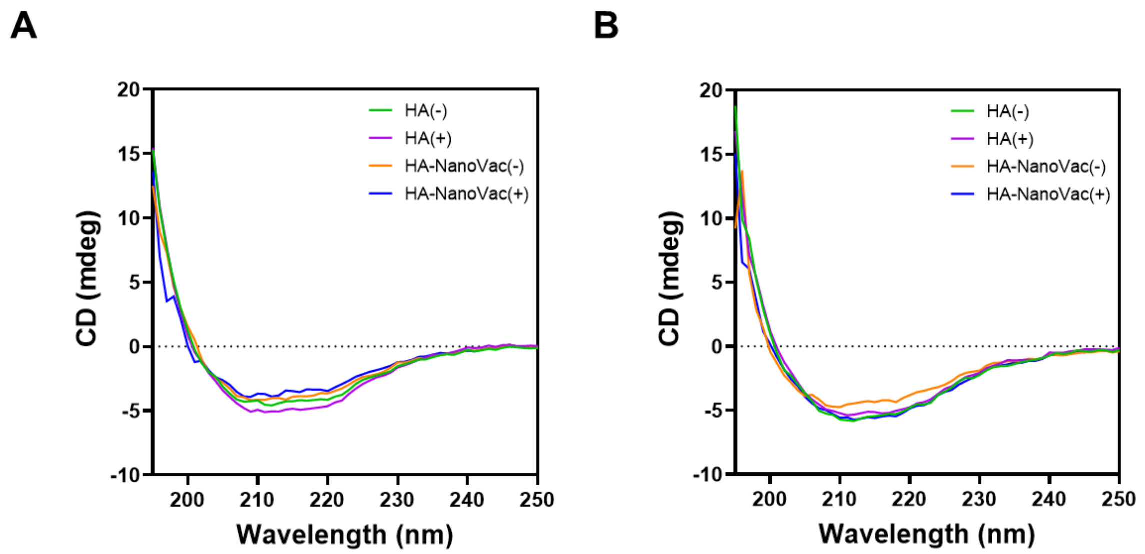

Figure S8. Circular dichroism (CD) spectra of HA and HA-NanoVac. A) CD spectra of HA and HA-NanoVac were measured in distilled water (DW). B) CD spectra of HA and HA-NanoVac were measured in PBS (pH 7.4). CD spectra of HA and HA-NanoVac were measured in presence (+) or absence (-) of laser irradiation (671nm, 1 J cm^{-2}).

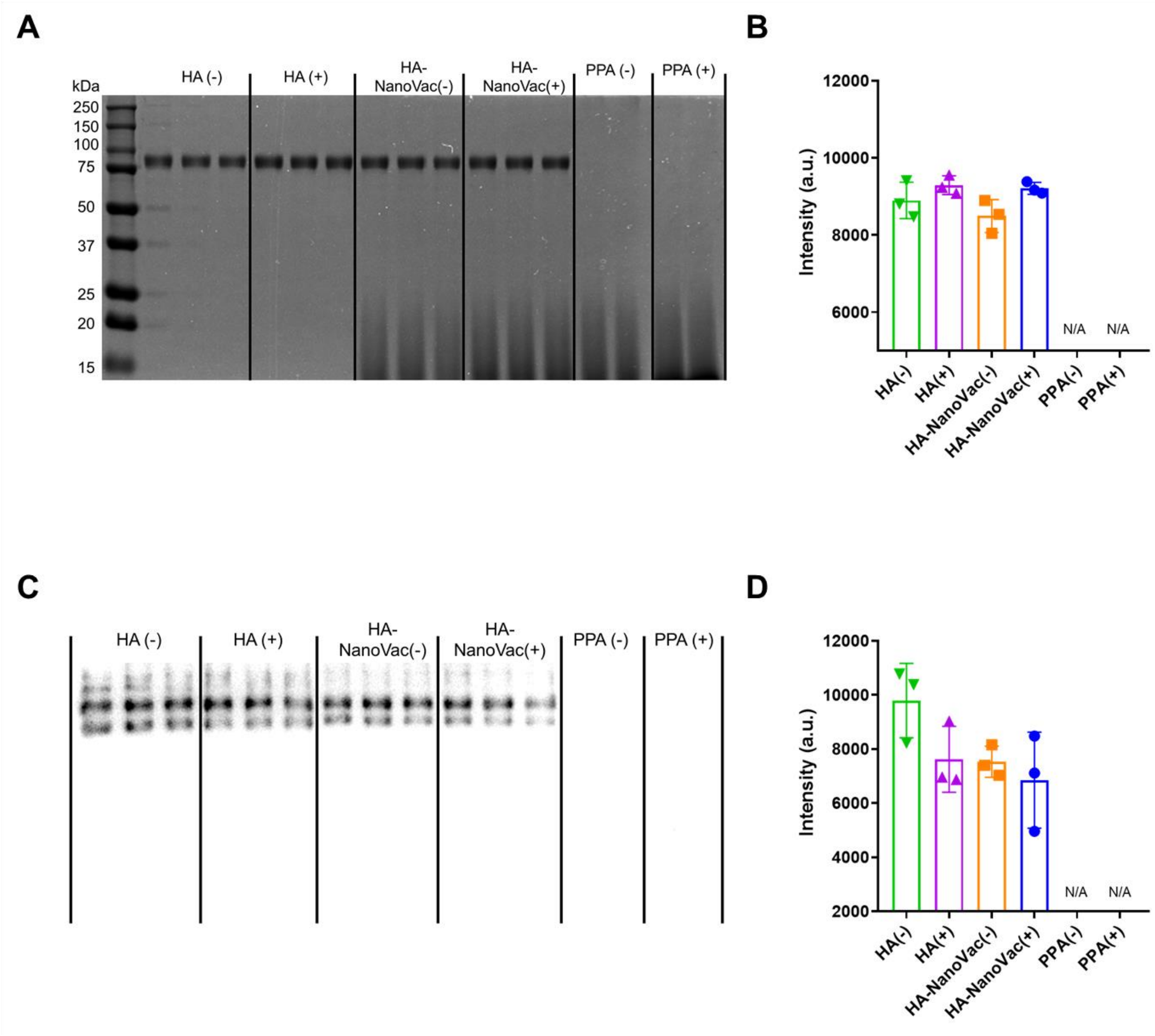

Figure S9. Preservation of protein characteristics via NanoVac formulations. A) SDS-PAGE of HA protein and HA-NanoVacs in with or without laser irradiation condition (1 J cm^{-2}). B) Protein band intensity analysis using Fiji/ImageJ program. C) Western blot of A). D) Band intensity analysis using ImageJ program. N/A, not available.

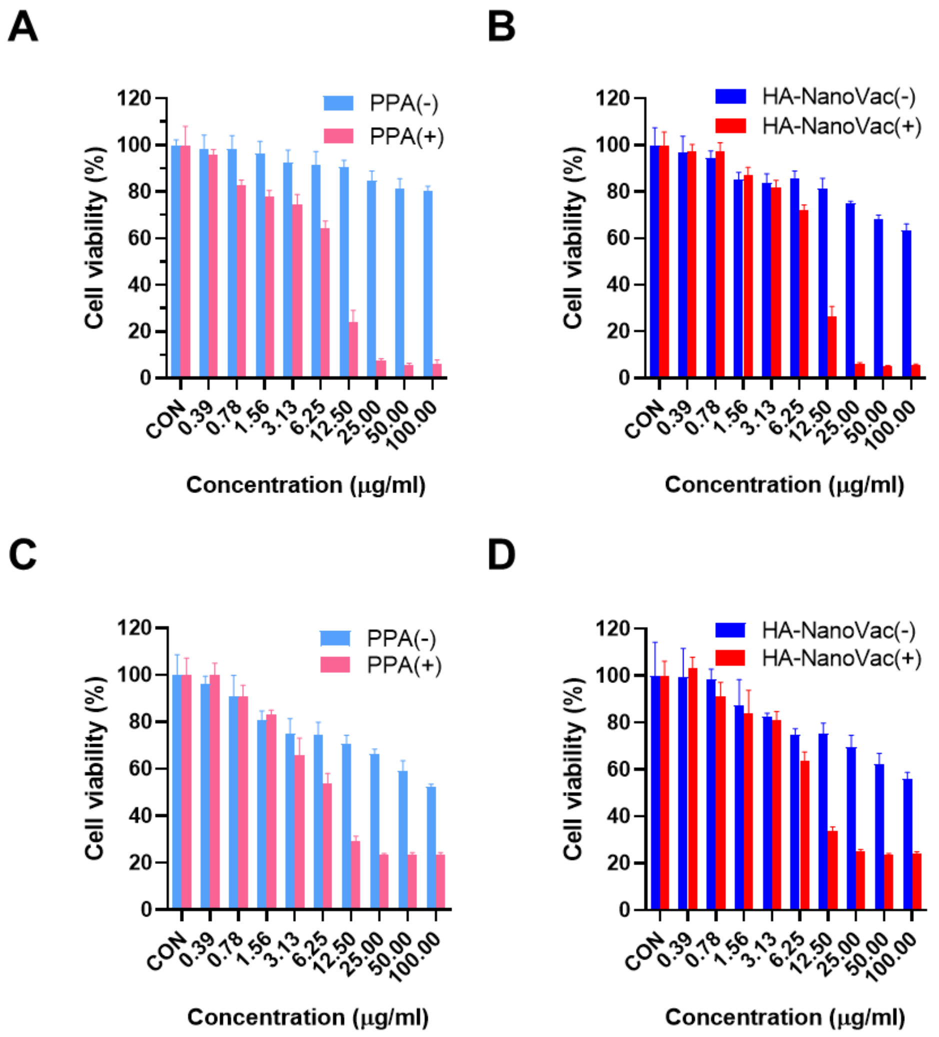

Figure S10. *In vitro* cytotoxicity of PPA and HA-NanoVac. A, C) Cytotoxicity of PPA with or without laser irradiation. B, D) Cytotoxicity of HA-NanoVac with or without laser irradiation. A-B) MTT assay was performed on MDCK cells (n=4, Mean ± SD). C-D) MTT assay was performed on WI38 cells (n=4, Mean ± SD). Laser irradiation; 670 nm, 1 J cm^{-2}.

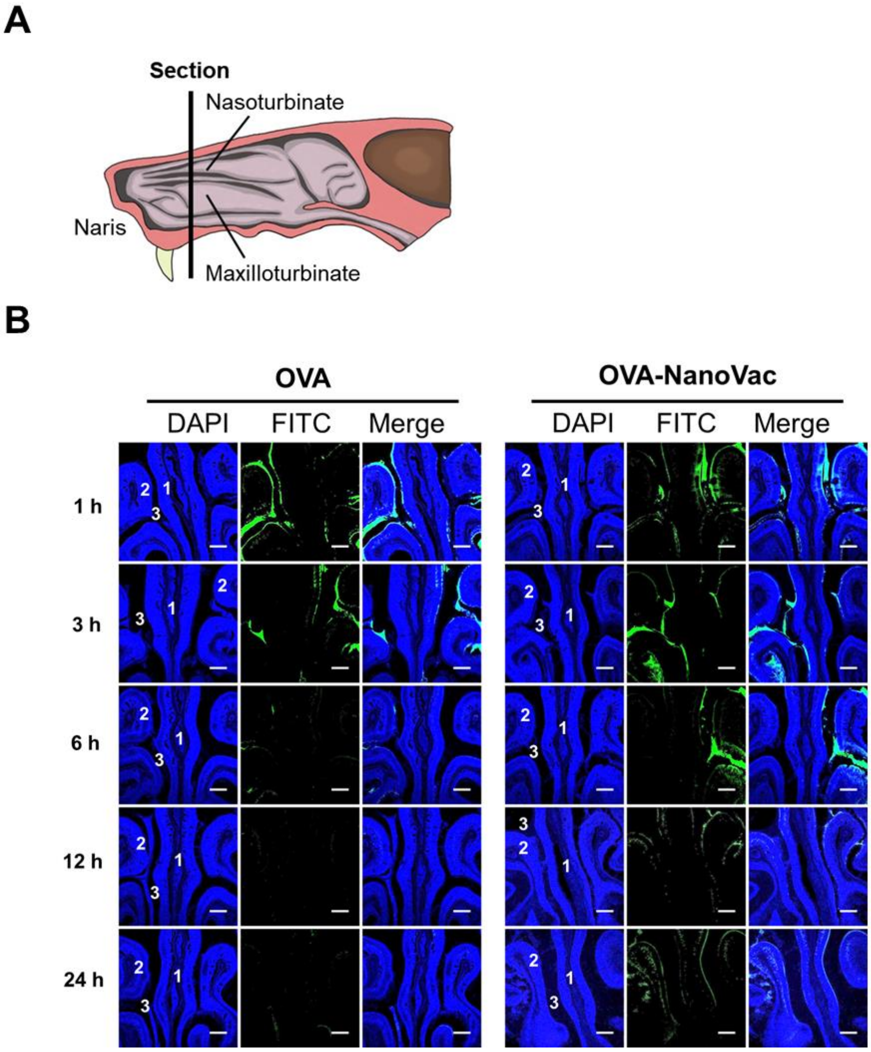

Figure S11. Histological illustration of sectional mice nasal region and enhanced intranasal delivery of OVA-NanoVacs via photochemical modulation. A) Anatomical illustration of mouse nose cavity and the incision site that used in the experiment. B) The OVA residence time in the nasal cavity was determined after intranasal administration of FITC-labeled OVAs (F-OVA) and F-OVA-NanoVacs. Fluorescence images of the nasal cavity of mice were observed by confocal laser scanning microscopy, Histological indication of mouse nasal cavity with white numbers (1-3). 1, nasal septum; 2, nasal meatus; 3, nasal turbinate. Scale bars, 200 μm.

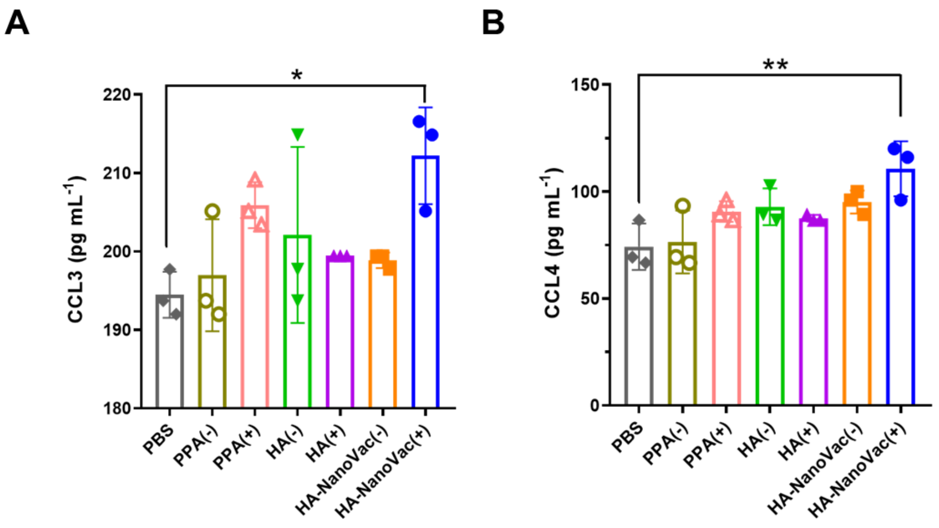

Figure S12. Photochemical immune induction of administration of NanoVac into nasal cavity. The CCL3 and CCL4 cytokine levels were detected in serum of the mice after immunization (PBS, PPA(-), PPA(+), HA(-), HA(+), HA-NanoVac(-), HA-NanoVac(+)). A) CCL3 and B) CCL4 levels in immunized with different samples. Data are presented as a mean value ± standard deviation (*$p<0.05$, **$p<0.01$). (BALB/c male, 6 weeks, n=3).

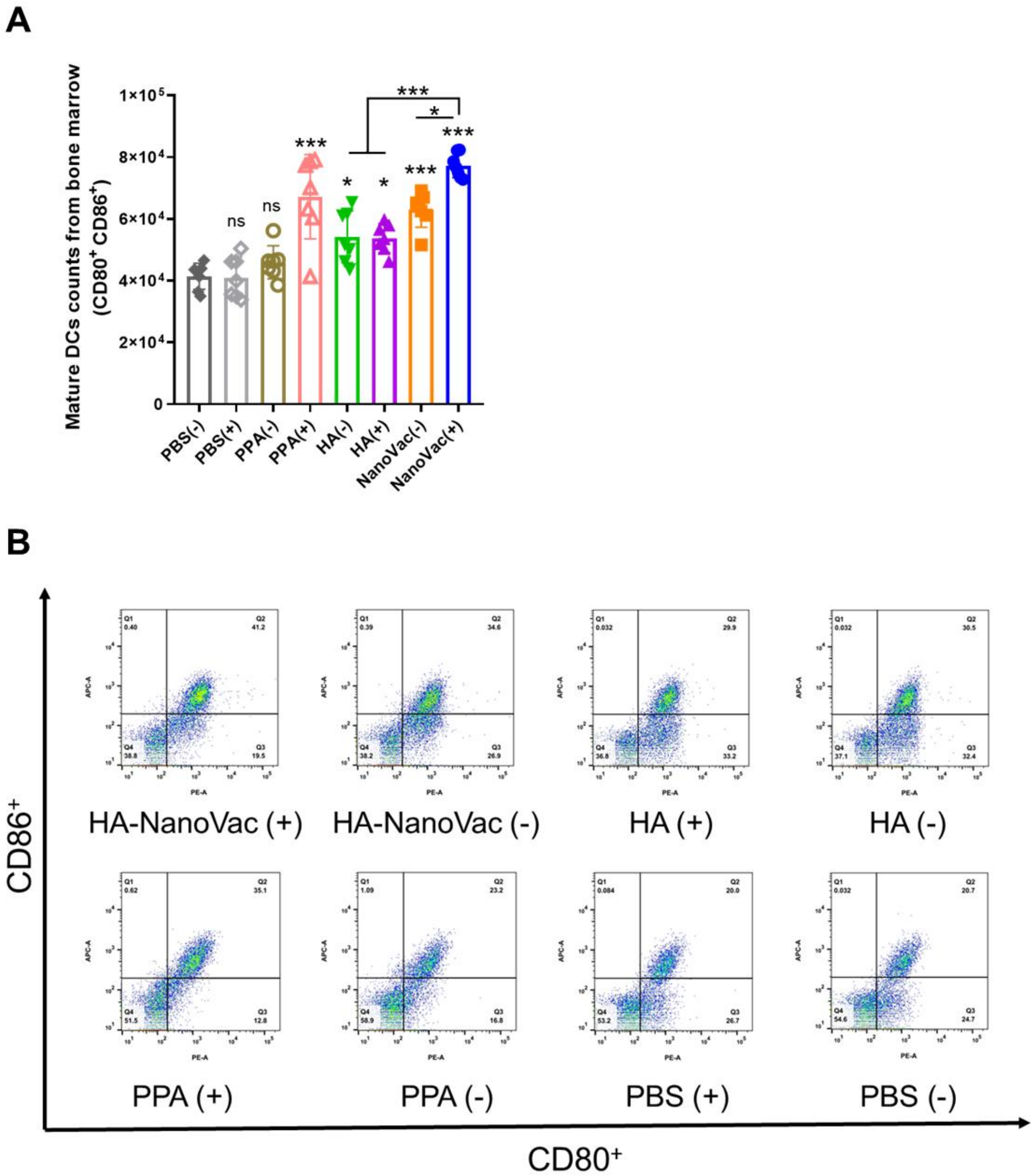

Figure S13. ***In vitro*** **maturation of dendritic cells (DCs) according to antigen protein treatment and laser irradiation.** The DCs derived from the bone marrow of mice (Balb/c male, 6 weeks). *In vitro* cultured BMDC maturation was confirmed with inoculation with PBS (-), PBS (+), PPA (-), PPA (+), HA (-), HA (+), HA-NanoVac (-) and HA-NanoVac (+). Data are presented as a mean value ± standard deviation (n=7, *p<0.05, ***p<0.001). (B) The data indicated flow cytometry profiles of the gating information used to identify $CD80^+$

and $CD86^+$ cells gated on $CD11c^+$ cells of sample treatment of cultured BMDC. Representative plot data were shown. (n=7)

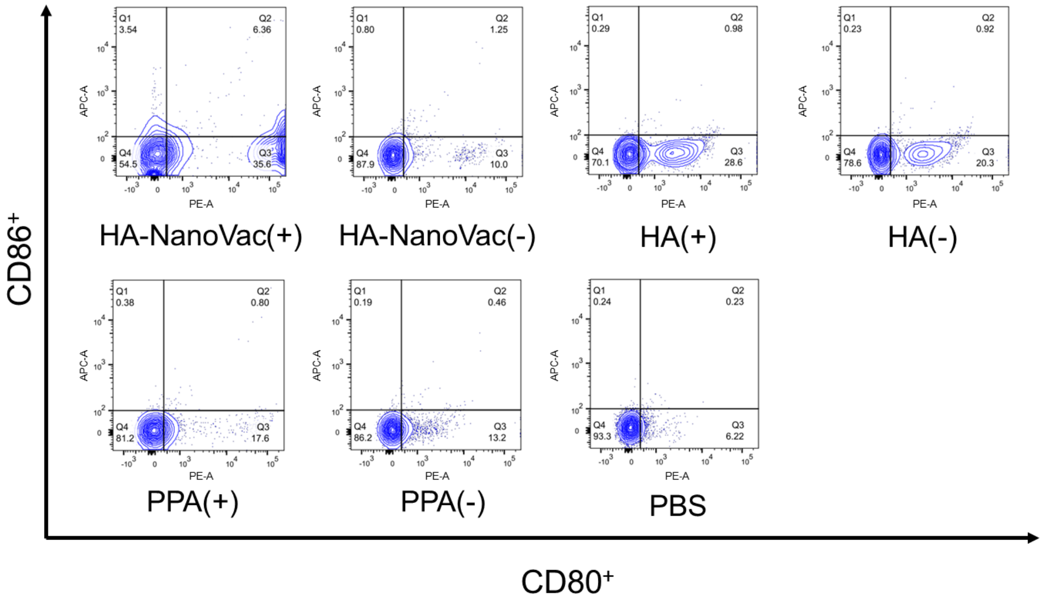

Figure S14. Confirmation of increased DCs maturation according to immunization and laser irradiation. The DCs maturation was confirmed in lymph nodes of the mice after immunization (PBS, PPA(-), PPA(+), HA(-), HA(+), HA-NanoVac(-), HA-NanoVac(+)). The data indicated flow cytometry profiles of the gating information used to identify CD80$^+$ and CD86$^+$ cells gated on CD11c$^+$ cells in lymph nodes. Representative plot data were shown. (BALB/c male, 6 weeks, n=3)

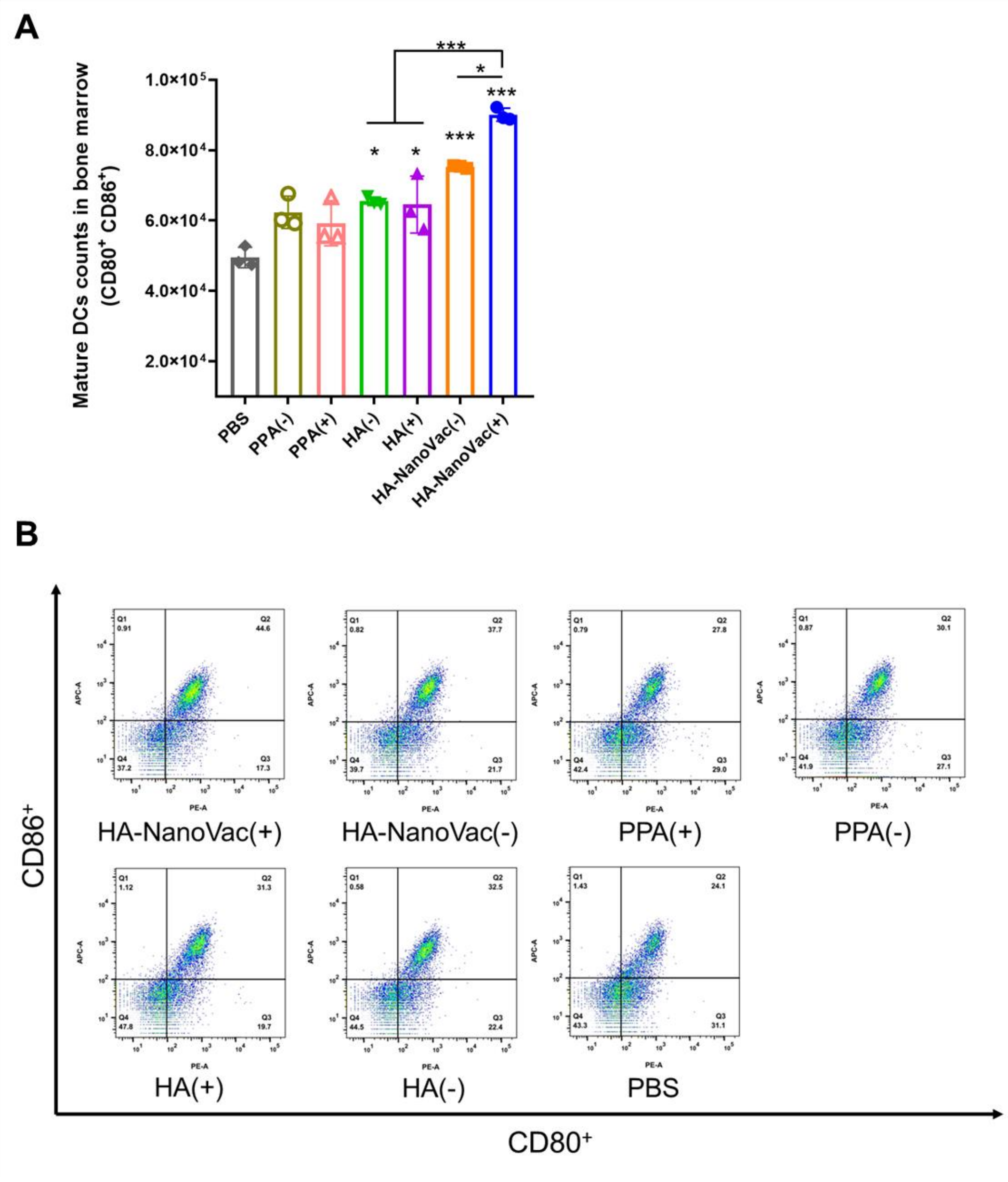

Figure S15. Confirmed maturation of bone marrow-derived dendritic cells (BMDCs) according to immunization and laser irradiation. A) The DCs maturation was confirmed that derived from the bone marrow of immunized mice (PBS, PPA(-), PPA(+), HA(-), HA(+), HA-NanoVac(-), HA-NanoVac(+)). B) The data indicated flow cytometry profiles of the gating information used to identify $CD80^+$ and $CD86^+$ cells gated on $CD11c^+$ cells in bone marrow. Representative plot data were shown. (BALB/c male, 6 weeks, n=3)

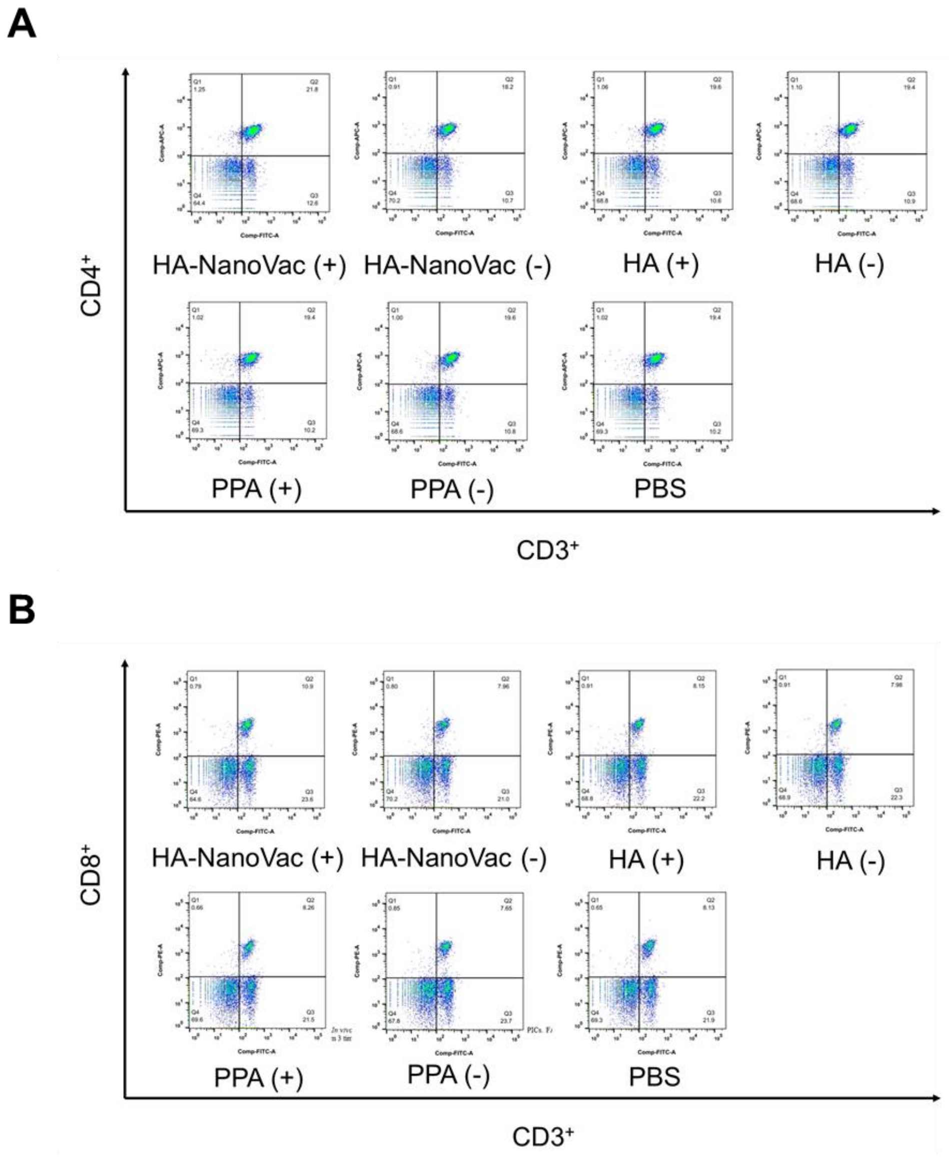

Figure S16. Confirmation of increased T cell population according to immunization and laser irradiation. T cell population was confirmed in the spleen of the mice after immunization (PBS, PPA(-), PPA(+), HA(-), HA(+), HA-NanoVac(-), HA-NanoVac(+)). The data indicated flow cytometry profiles of the gating information used to identify $CD3^+$/$CD4^+$ for $CD4^+$ helper T cells A) and $CD3^+$/$CD8^+$ for cytotoxic T cells B). Representative plot data were shown. (BALB/c male, 6 weeks, n=3)

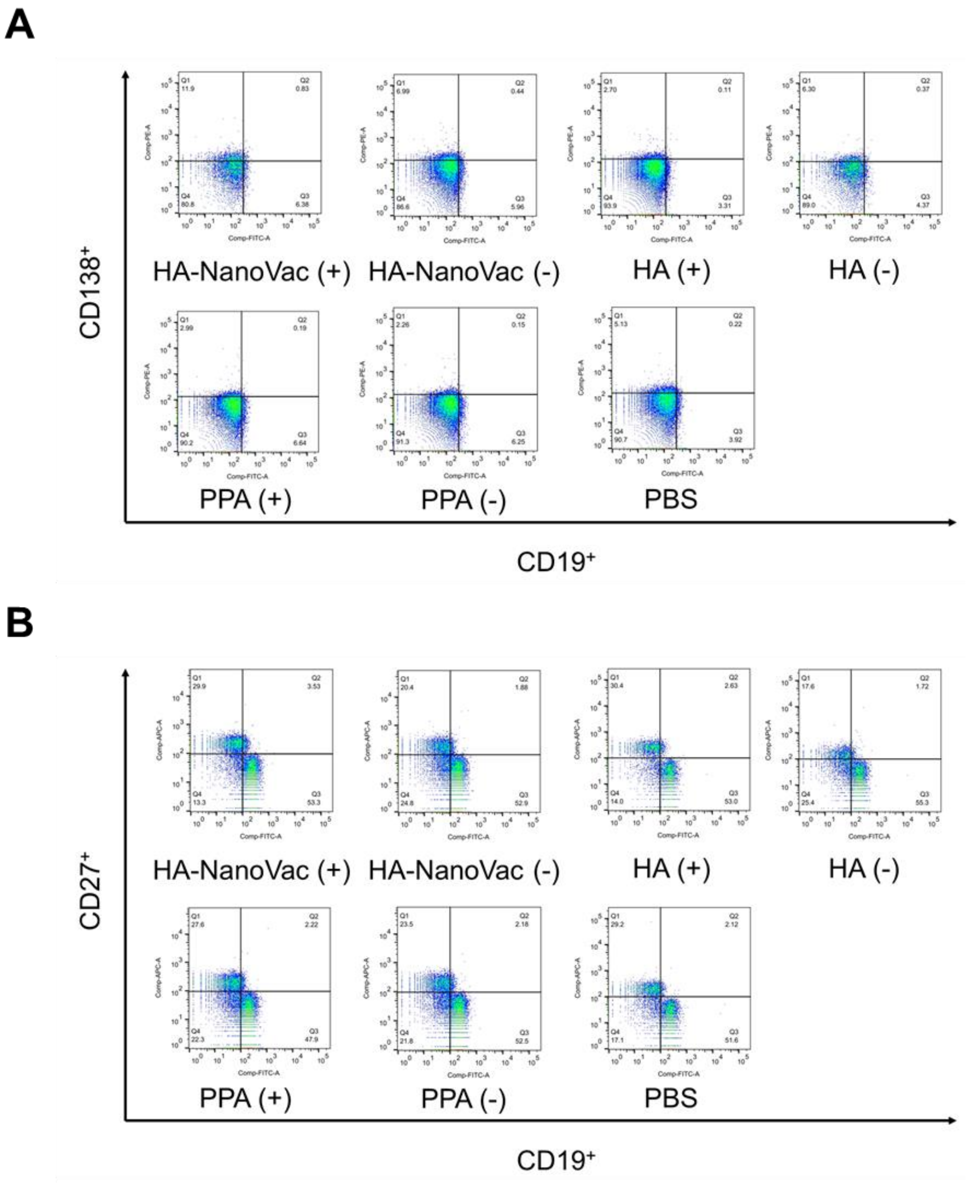

Figure S17. Confirmation of increased B cell population according to immunization and laser irradiation. B cell population was confirmed in the bone marrow of the mice after immunization (PBS, PPA(-), PPA(+), HA(-), HA(+), HA-NanoVac(-), HA-NanoVac(+)). The data indicated flow cytometry profiles of the gating information used to identify $CD19^-$/$CD138^+$ for plasma B cells A) and $CD19^+$/$CD27^+$ for memory B cells B). Representative plot data were shown. (BALB/c male, 6 weeks, n=3).

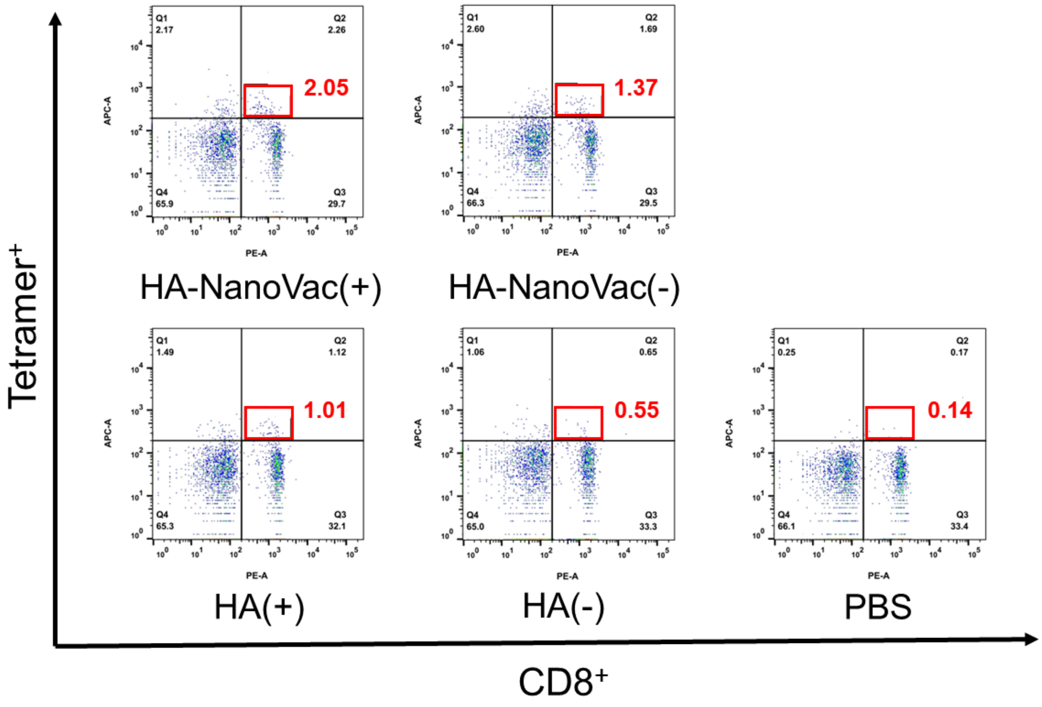

Figure S18. Confirmation of increased antigen-specific CD8 T^{+} cell population according to immunization and laser irradiation. T Antigen-specific CD8^{+} T cell population was confirmed using MHC tetramer (HA protein specific) in the spleen of the mice after immunization (PBS, HA(-), HA(+), HA-NanoVac(-), HA-NanoVac(+)). The data indicated flow cytometry profiles of the gating information used to identify CD8^{+} and Tetramer^{+} cells gated on CD3^{+} cells in spleen. Representative plot data were shown. (BALB/c male, 6 weeks, n=6)

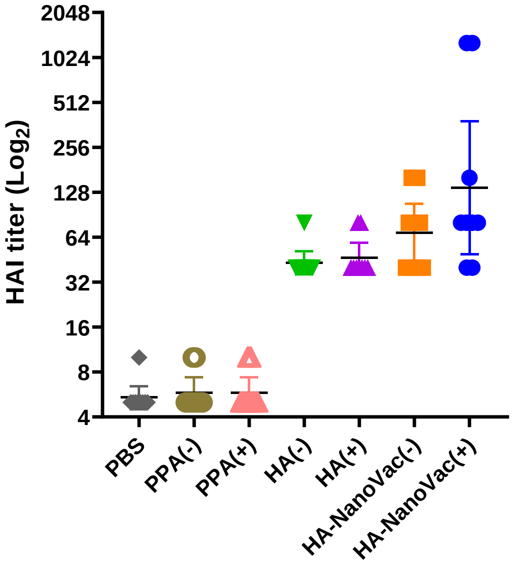

Figure S19. Hemagglutination inhibition (HAI) assay of sera in immunized mice. The horizontal lines indicate geometric mean titer (GMT) with 95% confidence intervals (CI). HAI assay was performed with pooled serum from each immunized group (BALB/c male, 6 weeks, n=8).

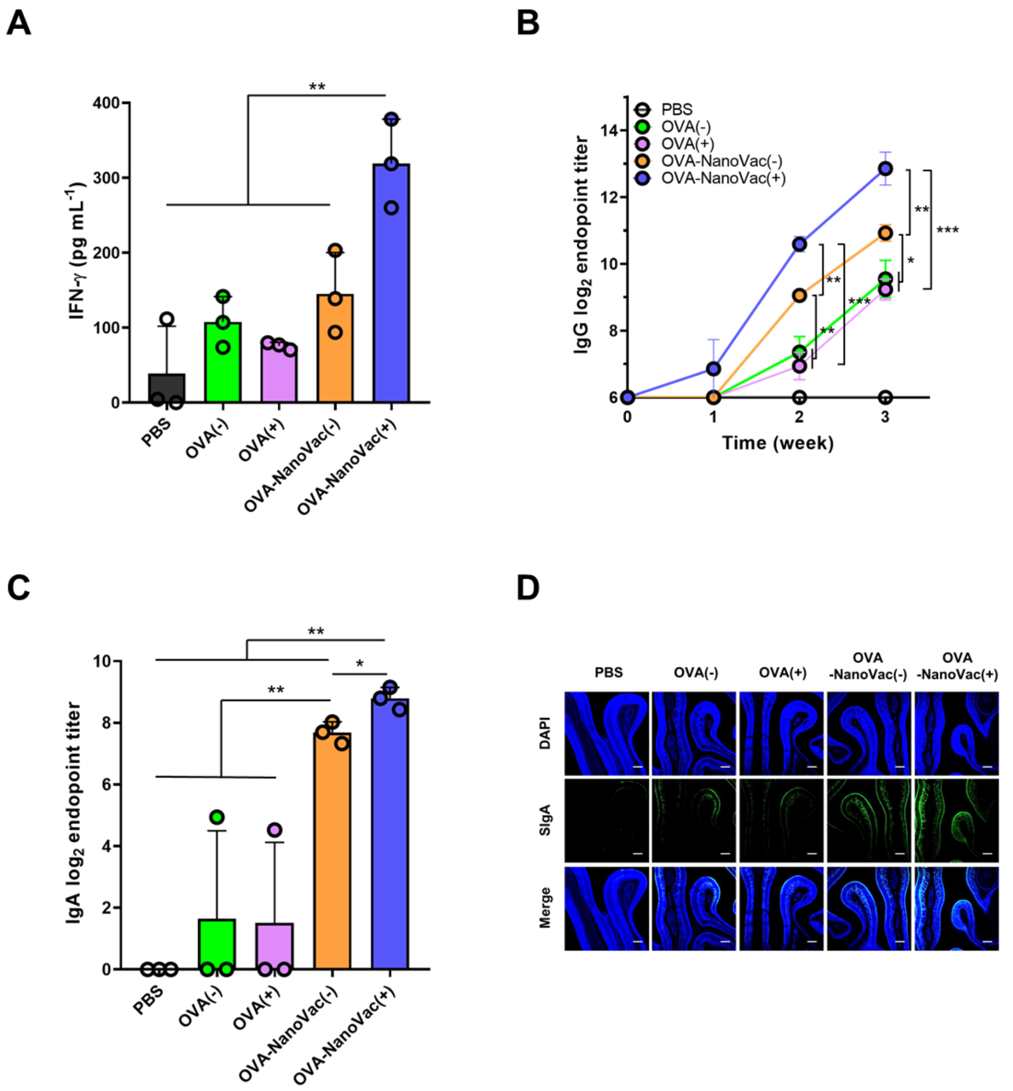

Figure S20. Photochemical induction of humoral and cellular immune responses to OVA. In the presence (+) or absence (-) of laser irradiation, A) the IFN-γ cytokine releasing was measured by ELISA from splenocytes of vaccinated mice. B) Anti-OVA IgG and C) IgA were measured by ELISA. D) SIgA in the nasal cavity were detected by confocal laser scanning microscope from immunized and laser irradiated mouse with each sample (Scale bars, 100 μm). Data are presented as a mean value ± standard deviation (*p<0.05, **p<0.01, ***p<0.001).

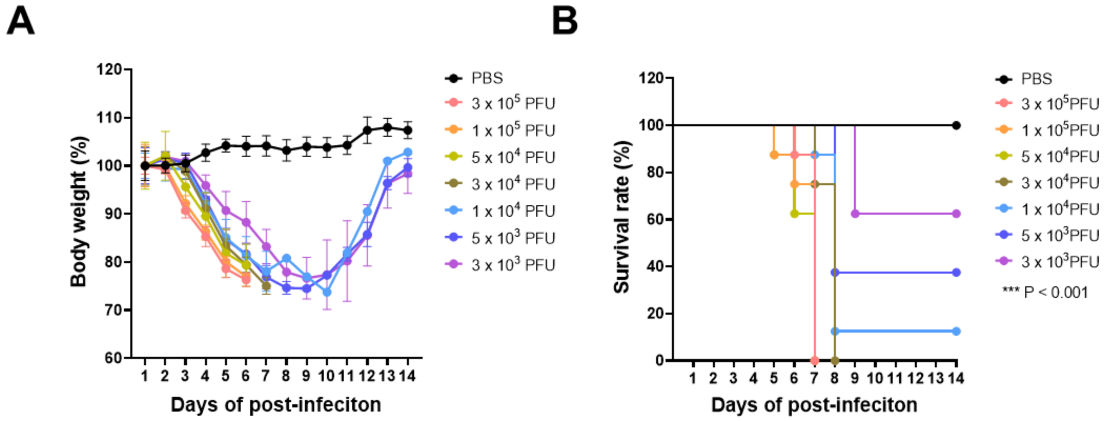

Figure S21. Determination of the 50% lethal dose (LD50) of influenza A virus in mice. Six-week-old male BALB/c mice (n=8) were infected intranasally with influenza virus (H1N1 A/California/07/2009, 3×10^{3}-3×10^{5} pfu mouse^{-1}). The change in A) body weights and B) survival rates were monitored for 14 days. The survival rates were determined by Reed-Muench method. Statistical significance in the survival rates was determined by the log-rank (Mantel-Cox) test in GraphPad Prism (*** P<0.001).

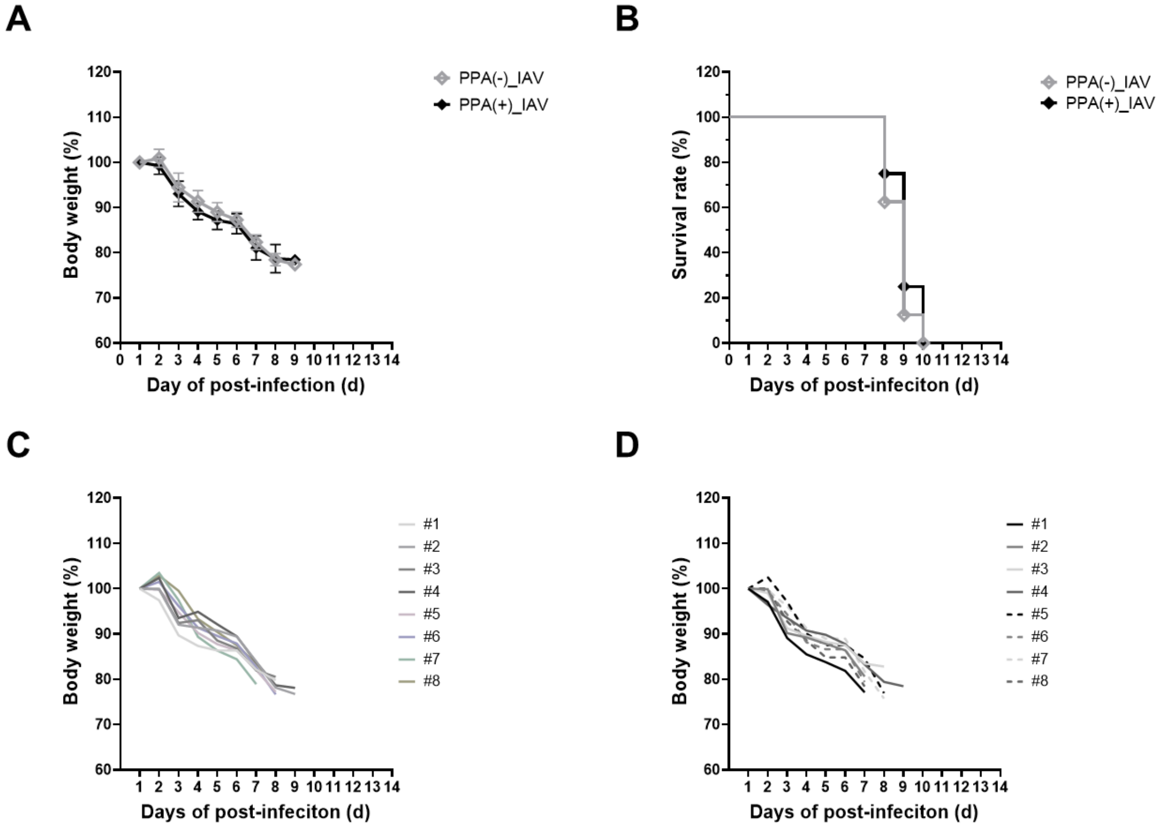

Figure S22. Influenza A virus challenge with PPA polymer inoculated mice. Mice were inoculated with PPA in the presence (+) and absence (-) of laser irradiation (BALB/c male, 6 weeks, n=8 mice per group). After 14 d of ended immunization, each mice group was challenged with influence A virus (15 LD50, 6×10^4 pfu/mouse). A) Body weight changes in 14 d after virus infection. B) Survival rates were monitored for 14 d. The statistical significance was indicated using the log-rank (Mantel-Cox) test (*** $P<0.001$, $P=0.0002$). Body weight changes per mouse in different immunization group against influenza virus A infection. C) PPA(-)_IAV, D) PPA(+)_IAV.

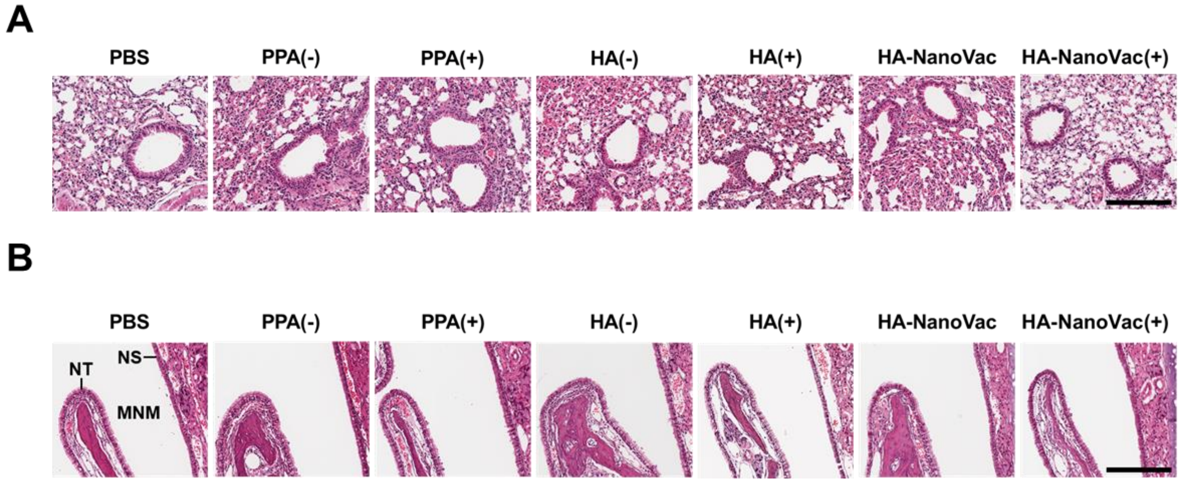

Figure S23. Histological images for confirmation in safety *in vivo*. Hematoxylin and eosin (H&E) staining images of immunized and laser irradiated mice. Mice were inoculated with PBS, PPA, HA and HA-NanoVac in the presence (+) or absence (-) laser exposure (50 J cm^{-2}, 670 nm). Histological images A) lung tissue and B) nasal cavities. B) NS, nasal septum; NT, nasal turbinate; MNM, middle nasal meatus. Scale bar, 200 μm.

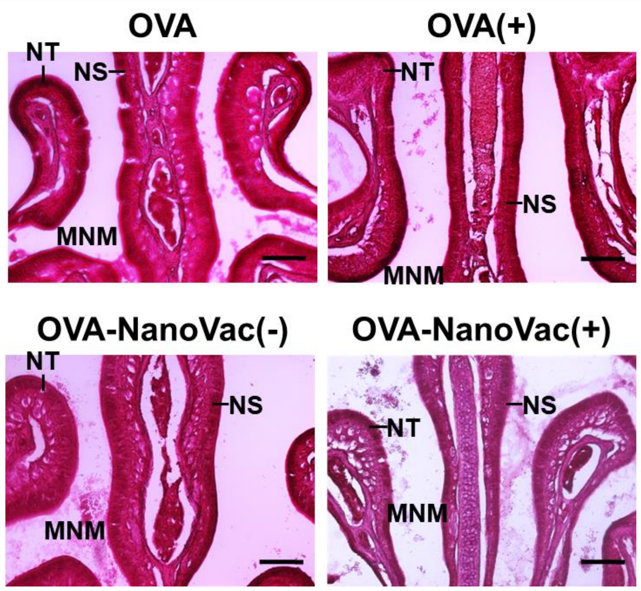

Figure S24. Histological images of nasal cavity and nasal epithelium of mice. Histological images of nasal cavity and nasal epithelium of mice at 7 days after administration of OVAs and OVA-NanoVac in the presence and absence of light irradiation. NS, nasal septum; NT, nasal turbinate; MNM, middle nasal meatus, Scale bars, 200 μm.

Table S1. Survival rate of mice against influenza A virus infection after immunization for each sample group.

		Survival rate of virus challenged after immunization (%)					
		PBS	PBS _IAV	HA(-) _IAV	HA(+) _IAV	HA-NanoVac(-) _IAV	HA-NanoVac(+) _IAV
Days of post-infection	1	100	100	100	100	100	100
	2	100	100	100	100	100	100
	3	100	100	100	100	100	100
	4	100	100	100	100	100	100
	5	100	100	100	100	100	100
	6	100	100	100	87.5	100	100
	7	100	87.5	87.5	87.5	100	100
	8	100	62.5	75	75	87.5	100
	9	100	12.5	32.5	37.5	62.5	100
	10	100	0	25	37.5	62.5	100
	11	100		25	37.5	62.5	100
	12	100		25	37.5	62.5	100
	13	100		25	37.5	62.5	100
	14	100		25	37.5	62.5	100

Table S2. Antibodies list of in this study

Name	Fluorescence	Company	Catalog No.
IgG	HRP	Bethyl	A90-116P
IgA	HRP	Bethyl	A90-103P
IgA	FITC	BD Pharmingen™	559354
CD3	FITC	BD Pharmingen™	555274
CD4	APC	BD Pharmingen™	553051
CD8a	PE	BD Pharmingen™	553033
CD11c	FITC	BD Pharmingen™	553801
CD19	FITC	BD Pharmingen™	553785
CD27	APC	BD Pharmingen™	560691
CD80	PE	BD Pharmingen™	553769
CD86	APC	BD Pharmingen™	558703
CD138	PE	BD Pharmingen™	553714
